# The Making of Calibration Sausage Exemplified by Recalibrating the Transcriptomic Timetree of Jawed Vertebrates

**DOI:** 10.3389/fgene.2021.521693

**Published:** 2021-05-12

**Authors:** David Marjanović

**Affiliations:** Department of Evolutionary Morphology, Science Programme “Evolution and Geoprocesses”, Museum für Naturkunde – Leibniz Institute for Evolutionary and Biodiversity Research, Berlin, Germany

**Keywords:** timetree, calibration, fossil record, gnathostomata, vertebrata, stemward slippage, divergence date

## Abstract

Molecular divergence dating has the potential to overcome the incompleteness of the fossil record in inferring when cladogenetic events (splits, divergences) happened, but needs to be calibrated by the fossil record. Ideally but unrealistically, this would require practitioners to be specialists in molecular evolution, in the phylogeny and the fossil record of all sampled taxa, and in the chronostratigraphy of the sites the fossils were found in. Paleontologists have therefore tried to help by publishing compendia of recommended calibrations, and molecular biologists unfamiliar with the fossil record have made heavy use of such works (in addition to using scattered primary sources and copying from each other). Using a recent example of a large node-dated timetree inferred from molecular data, I reevaluate all 30 calibrations in detail, present the current state of knowledge on them with its various uncertainties, rerun the dating analysis, and conclude that calibration dates cannot be taken from published compendia or other secondary or tertiary sources without risking strong distortions to the results, because all such sources become outdated faster than they are published: 50 of the (primary) sources I cite to constrain calibrations were published in 2019, half of the total of 280 after mid-2016, and 90% after mid-2005. It follows that the present work cannot serve as such a compendium either; in the slightly longer term, it can only highlight known and overlooked problems. Future authors will need to solve each of these problems anew through a thorough search of the primary paleobiological and chronostratigraphic literature on each calibration date every time they infer a new timetree, and that literature is not optimized for that task, but largely has other objectives.

## Introduction

This work is not intended as a review of the theory or practice of node (or tip) dating with calibration dates (or tip dates) inferred from the fossil record; as the most recent reviews of methods and sources of error, I recommend those by Barido-Sottani et al. (2019), Barido-Sottani et al. (2020), Marshall (2019), Matschiner (2019), Guindon (2020), Pardo et al. (2020), Powell et al. (2020), and, with caveats of which I will address two (see section “Materials and Methods”: Calibrations: Node 152 – Placentalia), Springer et al. (2019). Neither is it intended as a review of the history of the dates assigned to certain calibrations; as an example of a recent detailed review of three commonly used calibrations, I recommend Pardo et al. (2020). Although I discuss wider implications, the scope of this work is narrow: to evaluate each of the 30 calibrations used in the largest vertebrate timetree yet published, that by Irisarri et al. (2017), and the total impact of the errors therein on the results (using the same node-dating method they used, which I do not evaluate beyond mentioning potential general points of criticism).

Irisarri et al. (2017) inferred a set of timetrees from the transcriptomes of 100 species of gnathostomes (jawed vertebrates) and combinations of up to 30 calibrations from the fossil record. On the unnumbered ninth page of their supplementary information, they described their calibration dates as “five well-accepted fossil calibrations plus a prior on the root” and “24 additional well-established calibration points with solid paleontological evidence.” For many of the calibrations, these optimistic assessments are not tenable. I have tried to present, and use, the current state of knowledge on each of these calibrations.

In doing so, the present work naturally resembles the compendia of suggested calibrations that paleontologists have occasionally compiled with the intent to provide a handy reference for molecular biologists who wish to date divergences [e.g., Müller and Reisz, 2007; Benton et al., 2015, and six other articles in *Palaeontologia Electronica* 18(1); Wolfe et al., 2016; Morris et al., 2018]; Irisarri et al. (2017) took 7 of their 30 calibrations from the compendium in Benton and Donoghue (2007: table 1) alone—without citing the enlarged update by Benton et al. (2015)—compared to six taken from the primary literature. However, I will show that all such compendia are doomed to be (partially) outdated almost as fast as they are published in the best case, and faster than they are published in the average case. Soon, therefore, the present work will no longer be reliable as such a compendium either; rather, it is intended to show readers where the known uncertainties and disagreements lie, and thus what anybody who wants to use a particular calibration should probably search the most recent literature for. This is why I do not generally begin my discussion of a calibration by presenting my conclusions on what the best, or least bad, minimum and maximum ages of the calibration may be (They are, however, presented without further ornament in Table 1.) Instead, I walk the reader through a sometimes meandering discovery process, demonstrating how this knowledge was arrived at and how it may soon change—how the sausage was made and how it may spoil.

**TABLE 1:** The first four columns of Irisarri et al. (2017: supplementary table 8), here expanded to five, followed by the ages used here for the same calibrations and the differences (Δ).

Node number in Irisarri et al. (2017: supplementary table 8 and supplementary figure 19)	Description of cladogenesis	The sampled terminal taxa that stem from this node are:	Minimum age in Irisarri et al. (2017)	Maximum age in Irisarri et al. (2017)	Minimum age used here	Maximum age used here	Δ minimum ages	Δ maximum ages
100	Root node = Gnathostomata: total group including Chondrichthyes – Pan-Osteichthyes	Entire sample	421.75	462.5	465*	**475**	+43.25	+12.5
102	Osteichthyes: Pan-Actinopterygii – Sarcopterygii	Entire sample except Chondrichthyes	416	439	(420*)	(475)	+4	+36
104	Dipnomorpha – Tetrapodomorpha	Dipnoi – Tetrapoda	408	419	420*	(475)	+12	+56
105	Tetrapoda: Amphibia – Pan-Amniota	Lissamphibia – Amniota	330.4	350.1	335* (or 350*)	365	+4.6 (or +19.6)	+14.9
106	Amniota: Pan-Mammalia – Sauropsida	Mammalia – Reptilia	288	338	318*	(365)	+30	+27
107	Reptilia: Pan-Lepidosauria – total group of Archelosauria	Lepidosauria – Testudines, Crocodylia, Aves	252	257	(263*)	(365)	+11	+108
108	Archelosauria: Pan-Testudines – Pan-Archosauria	Testudines – Crocodylia, Aves	(243)	(257)	263*	(365)	+20	+108
109	Archosauria: Crocodylotarsi – Pan-Aves	Crocodylia – Aves	243	251	248*	252	+5	+1
111	Alligatoridae: Alligatorinae – Caimaninae	*Alligator* – *Caiman*	66	75	65*	200*	−1	+125
113	Neognathae: Galloanserae – Neoaves	*Anas*, *Gallus*, *Meleagris* – *Taeniopygia*	66	86.5	71	115	+5	+28.5
117	Testudines: Pan-Pleurodira – Pan-Cryptodira	*Phrynos*, *Pelusios* – all other sampled turtles	210	(257)	158*	**185**	−52	−72
124	Pleurodira: Pan-Chelidae – Pan-Pelomedusoides	*Phrynops* – *Pelusios*	25	(257)	125*	(185)	+100	−122
125	Lepidosauria: Rhynchocephalia – Pan-Squamata	*Sphenodon* – Squamata	238	(257)	244*	**290**	+6	+33
129	Toxicofera: Pan-Serpentes – Anguimorpha + Pan-Iguania	Snakes – their sister-group	“148” (165)	(257)	130*	(290)	“−18” (−35)	+33
131	Iguania: Pan-Acrodonta – Pan-Iguanidae	*Pogona*, *Chamaeleo* – *Iguana*, *Basiliscus*, *Sceloporus*, *Anolis*	165	230	72*	(290)	−93	+60
132	Iguanidae: Iguaninae + Corytophanidae – Phrynosomatidae + Dactyloidae	*Iguana*, *Basiliscus* – *Sceloporus*, *Anolis*	125	180	53*	(290)	−72	+110
150	Mammalia (Pan-Monotremata – Theriimorpha)	*Ornithorhynchus* – Theria	162.5	191.4	179*	233*	+16.5	+41.6
151	Theria: Metatheria – Eutheria	Marsupialia – Placentalia	124.6	138.4	126*	160	+1.4	+21.6
152	Placentalia: Atlantogenata – Boreo(eu)theria	*Loxodonta*, *Dasypus* – *Felis*, *Canis*, *Homo*, *Mus*	95.3	113	(66*)	72*	−29.3	−41
153	Boreo(eu)theria: Laurasiatheria – Euarchontoglires/Supraprimates	*Felis*, *Canis* – *Homo*, *Mus*	(61.5)	(113)	66*	(72*)	+4.5	−41
154	Carnivora: Pan-Feliformia – Pan-Caniformia	*Felis* – *Canis*	42.8	63.8	38*	56*	−4.8	−7.8
155	Euarchontoglires/Supraprimates: Gliriformes – Primatomorpha	*Mus* − *Homo*	61.5	100.5	65*	(72*)	+3.5	−28.5
157	Marsupialia: Didelphimorphia – Paucituberculata + Australidelphia	*Monodelphis* – *Macropus*, *Sarcophilus*	61.5	71.2	55*	68*	−6.5	−3.2
160	Batrachia: Urodela – Salientia	Caudata – Anura	249	(350.1)	249*	**290**	0	−60.1
169	Crown group of Cryptobranchoidea: Hynobiidae – Pancryptobrancha	*Hynobius* – *Andrias*	145.5	(350.1)	101*	(290)	−44.5	−60.1
170	Lalagobatrachia/Bombinanura: total group of Bombinatoroidea/Costata – total group of Pipanura	*Bombina*, *Discoglossus* – all other sampled frogs	161.2	(350.1)	(153*)	(290)	−8.2	−60.1
171	Pipanura: total group of Pipoidea/Xenoanura – total group of Acosmanura	*Pipa*, *Hymenochirus*, *Silurana* – their sister-group	145.5	(350.1)	153*	(290)	+7.5	−60.1
178	Pipidae: Pipinomorpha – Xenopodinomorpha	*Pipa* – *Silurana*, *Hymenochirus*	86	(350.1)	84*	199*	−2	−151.1
187	Crown group of Chondrichthyes (Holocephali – Elasmobranchii)	*Callorhinchus* – Elasmobranchii	410	“495” (462.5)	385*	(475)	−25	“-20” (+12.5)
188	Crown group of Elasmobranchii (Selachimorpha – Batomorpha)	Sharks – rays	190	(462.5)	201*	395*	+11	−67.5
192	Batoidea (Rajiformes – rays)	*Neotrygon* – *Raja*, *Leucoraja*	176	(462.5)	184*	201*	+8	−261.5
195	Neopterygii (Holosteomorpha – Pan-Teleostei)	*Lepisosteus*, *Amia* – *Takifugu*, *Danio*	345	392	249*	299	−96	−93

**FIGURE 1:**
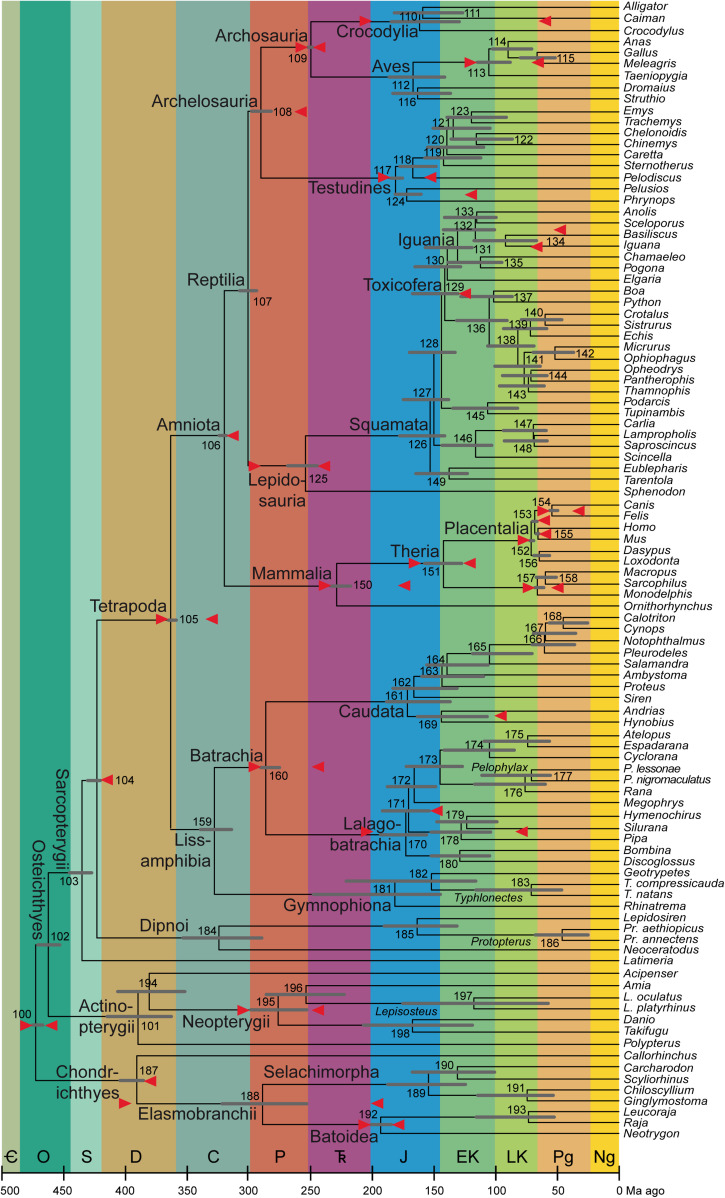
Average timetree resulting from application of the calibrations described here (mostly in the Supplementary Material). As in Table 2 and in Irisarri et al. (2017: figure 3), the bars on the nodes are the superimposed 95% credibility intervals from the two runs in PhyloBayes. The calibrations are shown as red arrows horizontally in line with the nodes they apply to; note that the arrow that is almost aligned with the branch of Lalagobatrachia and the one that is almost aligned with the terminal branch for *Silurana* are the maximum and minimum ages of Node 178 (Pipidae), the one on *Iguana* to Node 131 (Iguania), and the one on *Pelodiscus* to Node 117 (Testudines). The abbreviated genus names are spelled out as clade names on their common branches; where only one species per genus is sampled, see Irisarri et al. (2017) for full species names. To the extent possible, clade names with minimum-clade (node-based) definitions are placed close to those nodes, while names with maximum-clade (branch-based) definitions are shown close to the origin of that branch (i.e., the preceding node if sampled) and undefined names stay in the middle. Period/epoch symbols from oldest to youngest: Cambrian (cut off at 500 Ma), Ordovician, Silurian, Devonian, Carboniferous, Permian, Triassic, Jurassic, Early Cretaceous, Late Cretaceous, Paleogene, and Neogene including Quaternary (which comprises the last 2.58 Ma and is not shown separately). Timescale (including colors) from the International Chronostratigraphic Chart, version 2020/03 (Cohen K. M. et al., 2020). Node numbers, also used in the text and the tables, are from Irisarri et al. (2017).

*Boldface is a rough indicator of my confidence. Hard bounds are marked with an asterisk (though all nodes had to be treated as hard in my analysis, see text). Dates in parentheses were not specified in the analysis; the node was constrained in practice by the given constraint on a preceding (for maximum ages) or following node (for minimum ages) elsewhere in this table—see Figure 1 for which nodes precede each other. The two dates in quotation marks were specified by Irisarri et al. (2017), but had no effect because they were in practice constrained by the dates specified for other nodes. Dashes in the second and third column separate the two branches stemming from the node in question. Depending on the node, see the text or the Supplementary Material for discussion and references.*

Some works used as compendia in this sense are not even compiled by paleontologists: molecular biologists often copy from each other. Irisarri et al. (2017) took four of their calibrations from table 1 of Noonan and Chippindale (2006), a work that contains a phylogenetic and divergence-date analysis of molecular data and cites severely outdated paleontological primary and secondary literature (from 1981 to 2003) as its sources.

A continually updated online compendium could largely avoid the problem that knowledge has a half-life. There has been one attempt to create one, the Fossil Calibration Database (Ksepka et al., 2015^1^; not counting separately its predecessor, called Date a Clade, which is no longer online and apparently merely presented Table 1 of Benton and Donoghue, 2007). It appears to have run out of funding long ago and has not been updated since February 2, 2018, the day on which three of the numerous calibrations proposed in Wolfe et al. (2016) were added; other calibrations from the same source were added on January 30 and 31, 2018 (one each) and December 22, 2017 (three), and no other updates were made on those days. I cannot resist pointing out that this is one of many cases where funding menial labor in the sciences—reading and interpreting papers, evaluating the contradictions between them, and entering the interpretations in a database, a task that cannot be automated—would go a long way toward improving the quality of a large number of publications, but is unlikely to be granted because it is not likely to result in a single flashy publication or in an immediately marketable application directly, even though precise and accurate timetrees are an essential component of our understanding of the model organisms used in biomedical research.

A continually updated online database aiming to represent the entire fossil record exists and is currently being funded: the Paleobiology Database, accessible through two different interfaces at http://www.pbdb.org and https://paleobiodb.org. Among many other things, it aims to contain the oldest currently known record of every taxon and would thus be useful as a source for calibrations. However, the warnings by Parham et al. (2011) still apply: the quality of the Paleobiology Database is quite heterogeneous. While some entries are written by the current top experts in the respective fields, others copy decades-old primary descriptive literature uncritically, often leading to severely outdated taxonomic, let alone phylogenetic placements (in all but the most recent literature that is not the same), not to mention misunderstandings based on the convoluted history of taxonomic nomenclature. It is not uncommon for two entries to contradict each other. Finally, despite the hundreds of contributors, our current knowledge of the fossil record is so vast that the database remains incomplete (again, of course, differently so for different taxa). Like Irisarri et al. (2017), I have not used the Paleobiology Database or the Fossil Calibration Database; I have relied on the primary literature.

### Nomenclature

After the publication of the *International Code of Phylogenetic Nomenclature (PhyloCode)* (Cantino and de Queiroz, 2020) and its companion volume *Phylonyms* (de Queiroz et al., 2020), the registration database for phylogenetic nomenclature—*RegNum* (Cellinese and Dell, 2020)—went online on June 8, 2020; regulated phylogenetic nomenclature is therefore operational. In an effort to promote uniformity and stability in nomenclature, I have used the names and definitions from *Phylonyms*, Ezcurra et al. (2020: online methods) and Joyce et al. (2021) here; wherever applicable, all of them are followed by “[PN]” at least at the first mention (this includes vernacularized forms like “gnathostome”) to avoid confusion with earlier uses of the same names for different clades. I have not, however, followed the *ICPN*’s Recommendation 6.1A to set all taxonomic names in italics.

The definitions of these names, their registration numbers (which establish priority among the combinations of name and definition), and the exact chapter citations can be found in *RegNum*, which is freely accessible^2^.

*ICPN*-regulated names have not been created or converted according to a single overarching scheme. As a result, for example, the name Osteichthyes has been defined as applying to a crown group, and the corresponding total group has been named Pan-Osteichthyes, but the name Chondrichthyes has not been defined and could end up as the name for a crown group, a total group, or neither (indeed, current common usage by paleontologists is neither). This has required some awkward circumlocutions. Following Recommendation 9B of the *ICPN*, I have not coined any new names or definitions in the present work.

The shapes and definitions of most other taxonomic names used here do not currently compete for homonymy or synonymy under any code of nomenclature. (The *ICPN* is not retroactive, and the rank-based *International Code of Zoological Nomenclature* [International Commission on Zoological Nomenclature, 1999] does not regulate the priority of names at ranks above the family group.) In such cases, I have followed current usage where that is trivial; I occasionally mention synonyms where that seems necessary.

The usage of “stem” and “crown” requires a comment. The crown group of a clade consists of the last common ancestor of all extant members of that clade, plus all its descendants. The rest of the clade in question is its stem group. For example, *Gallus* is a crown-group dinosaur, and *Triceratops* is a stem-group dinosaur. In a development that seems not to have been foreseen by the first two or so generations of phylogeneticists that established the terminology—for example, the zoology textbook by Ax (1987) exclusively named total groups, i.e., halves of crown groups!—many clades with defined names are now identical to their crown groups (in other words, they are crown clades); they do not contain any part of their stem. Aves [PN] is an example; although *Triceratops* is a stem-dinosaur [PN], a stem-dinosauromorph [PN], and a stem-ornithodiran [PN] among other things, it is not a stem-bird or stem-avian because by definition there is no such thing. It is instead a stem-pan-avian [PN], i.e., a stem-group member of Pan-Aves [PN] (Ezcurra et al., 2020: online methods). If no name is available for a suitable larger group, I have resorted to the circumlocution that *Triceratops*, for instance, is “on the bird stem” or “in the avian total group” (expressing that it is closer to Aves than to any mutually exclusive crown group).

## Materials and Methods

Although I have followed the spirit of the guidelines developed by Parham et al. (2011) for how best to justify or evaluate a proposed calibration, I have not consistently followed their letter. Most notably, the specimen numbers of the fossils that I largely refer to by genus names can all be found in the directly cited primary literature, so they are not repeated here.

### Hard and Soft Minima and Maxima

Without discussing the matter, Irisarri et al. (2017) stated that they had treated all calibration ages as soft bounds, which, in the software they used, means that “a proportion of 0.05 of the total probability mass is allocated outside the specified bound(s) (which means, 5% on one side, in the cases of the pure lower and pure upper bounds, and 2.5% on each side in the case of a combination of lower and upper bound)” (Lartillot, 2015: manual). This is particularly odd for minimum ages; after all, the probability that a clade is younger than its oldest fossil is not 5% or 2.5%, it is 0%. A few other works have used soft minima as an attempt to account for phylogenetic or chronostratigraphic uncertainty of the specimens chosen as calibrations. I have not used the former approach here (despite two clumsy attempts in the first preprint of this paper—Marjanović, 2019—that were rightly pointed out as incoherent by a reviewer): in the cases of phylogenetic uncertainty discussed below, different fossils that could calibrate the age of a cladogenetic event are commonly tens of millions of years apart, a situation that cannot be smoothed over by using the oldest one as a soft minimum. Soft minima that can be justified by uncertainty over the exact age of a calibrating fossil are very rare nowadays (as already pointed out by Parham et al., 2011); within the scope of this paper, there is only one such case, the minimum age of Neognathae (node 113), which is determined by a specimen that is roughly 70 ± 1 Ma old according to a fairly long chain of inference. I have treated all other minima as hard, and I have not spelled this out below.

As recommended by Parham et al. (2011), minimum ages have generally been chosen in the literature as the youngest possible age of the calibrating specimen(s). This is practically guaranteed to result in ages that are too young for various reasons (Marshall, 2019). To account, if crudely, for non-zero branch lengths and especially for the nested phylogenetic positions of some calibrating specimens, and to counteract “the illusion of precision” (Graur and Martin, 2004: title) spread by calibration ages with five significant digits like 421.75 Ma [the minimum age chosen by Irisarri et al. (2017) for the root node, see below], I have rounded up (stratigraphically down) to the nearest million years, with a few exceptions suggested by mass extinction events.

Maximum ages are by default much more difficult to assign than minimum ages. Absence of proof is not proof of absence; absence of evidence is evidence of absence, but in most cases it is quite weak evidence. Yet, omitting maximum ages altogether and assigning only minimum ages to all calibrations automatically results in much too old divergence dates as nothing stops the 99.9% or 99.99% confidence or credibility intervals for all node ages from avoiding all overlap with the calibrated minimum ages. I have therefore followed Irisarri et al. (2017) and their sources in assigning as many maximum ages as I dare. For this purpose, I have basically followed the recommendations of Parham et al. (2011) and Pardo et al. (2020: 11), which amount to assigning a maximum age whenever we can reasonably expect (after preservation biases, collection biases, collection intensity, paleobiogeography, etc.) to have found evidence of the clade in question if it had been present at the time in question, but have not found any. This has widely been followed in the literature, but various compendia like Benton et al. (2015) have gone beyond this in many cases: in short, the oldest certain fossil provides the minimum age under that approach, while the oldest uncertain fossil of the same clade provides the maximum age. This practice is not defensible; therefore, I assign, in the aggregate, fewer and more distant maximum ages than Irisarri et al. (2017).

Given the limits of our current knowledge of the fossil record, all maximum ages might be expected to be soft bounds. In a few cases discussed below, however, I find that the absence of evidence is so hard to explain away that a hard maximum is justified. This generally concerns unrealistically old maxima that I have chosen because no younger maximum suggests itself. Ultimately, of course, this is subjective.

The choices of hard vs. soft bounds do not seem to make a great difference to the big picture. Due to practical constraints, a set of calibration ages mostly identical to the present ones was analyzed twice, with all bounds treated as soft or as hard, in the first preprint of this work (Marjanović, 2019); the results were quite similar to each other (Marjanović, 2019: figure 1 and table 2). Even so, however, in the run where all bounds were soft, most divergence dates were younger than in the run where all bounds were hard (usually negligibly so, but by 20 Ma in the extreme cases); the mean ages of some calibrated nodes even ended up younger than their minimum ages.

### Calibrations

Because this journal imposes a space restriction, most of this section forms the Supplementary Material.

In the nine subsections below and the 20 sections of the Supplementary Material, I discuss the minimum and maximum ages of all 30 nodes used as calibrations by Irisarri et al. (2017), referring to each by clade names and by the node number assigned by Irisarri et al. (2017: especially supplementary table 8 and supplementary figure 19), also shown in Figure 1. The abbreviation Fm stands for Formation; ICSC refers to the International Chronostratigraphic Chart v2020/3 (Cohen K. M. et al., 2020); Ma is the quasi-SI symbol for megayear (million years).

#### Root Node (100): Gnathostomata [PN] (Total Group Including Chondrichthyes – Pan-Osteichthyes [PN])

The cladogenesis that created the total groups of Chondrichthyes and Osteichthyes [PN] was assigned a minimum age of 421.75 Ma, a remarkably precise date close to the Silurian-Devonian boundary, and a maximum age of 462.5 Ma, which is currently (ICSC) thought to lie in the Darriwilian stage of the Middle Ordovician.

The Darriwilian should rather be regarded as the minimum age of this calibration date. While articulated bones and teeth of gnathostomes—both total-group chondrichthyans (Burrow and Young, 1999) and pan-osteichthyans (Choo et al., 2017, and references therein)—are only known from the Ludfordian (Ludlow, late Silurian) upward, a large diversity of scales that are increasingly confidently assigned to stem-chondrichthyans extends all the way down into the early Darriwilian (Sansom et al., 2012; Andreev et al., 2015, 2016a, b; Sansom and Andreev, 2018; Žigaitė-Moro et al., 2018; and references therein). The Darriwilian is currently thought to have begun 467.3 ± 1.1 Ma ago and to have ended 458.4 ± 0.9 Ma ago (ICSC); for the purposes of reducing “the middle part of the Stairway Sandstone” (Sansom et al., 2012, p. 243) to a single number, the age of 465 Ma should be adequate as the minimum age of Gnathostomata.

As a maximum age, I cautiously propose the mid-Floian (Early Ordovician) upper fossiliferous level of the Burgess-like Fezouata Shale; at both levels, gnathostomes are absent among the “over 200 taxa, about half of which are soft-bodied” (Lefebvre et al., 2017, p. 296). Note that the oldest known hard tissues of vertebrates are Floian in age as well (reviewed by Sansom and Andreev, 2018). The Floian began 477.7 ± 1.4 Ma ago and ended 470.0 ± 1.4 Ma ago (ICSC), so I suggest a soft maximum age of 475 Ma for this calibration date.

The minimum and the maximum age proposed here are unexpectedly close together. This may be a sign that one or both is an unduly optimistic assessment of our knowledge of the fossil record—or that the origin of Gnathostomata formed part of the Great Ordovician Biodiversification Event (Sansom et al., 2012; Sansom and Andreev, 2018), which does not seem implausible.

#### Node 105: Tetrapoda [PN] (Amphibia [PN] – Pan-Amniota [PN])

The divergence between the ancestors of lissamphibians and those of amniotes was assigned a minimum age of 330.4 and a maximum of 350.1 Ma following Benton and Donoghue (2007). Although Pardo et al. (2020) have reviewed the breadth of issues it (many raises far beyond the scope of this work), and although I broadly agree with their conclusions, a few points remain to be addressed or summarized.

For a long time, the oldest tetrapod was thought to be *Lethiscus*, variably supposed to be a stem-amphibian or a stem-pan-amniote (see below), which is mid-Viséan in age (Smithson et al., 2012, and references therein; the Viséan lasted from 346.7 ± 0.4 to 330.9 ± 0.2 Ma ago: ICSC). More likely, *Lethiscus* and the other aïstopods are rather early-branching stem-stegocephalians [PN] (Pardo et al., 2017, 2018; Clack et al., 2019; further discussion in Marjanović and Laurin, 2019). Whether *Casineria* from a geographically (southeastern Scotland) and stratigraphically close site (mid-late Viséan: Paton et al., 1999; Smithson et al., 2012) can replace it in that function depends on two unresolved issues: its own phylogenetic position, for which estimates range from very close to Amniota (within Tetrapoda) into Temnospondyli (Marjanović and Laurin, 2019, and references therein; Clack et al., 2019; Daza et al., 2020: supplementary figure S15), and the controversial phylogenetic position of Lissamphibia [PN] in the stegocephalian tree (Marjanović and Laurin, 2013a, 2019; Danto et al., 2019; Laurin et al., 2019; Daza et al., 2020; Pardo et al., 2020; and references in all five), which determines whether the temnospondyls are tetrapods or quite rootward stem-stegocephalians by determining which node of the otherwise largely stable tree of early stegocephalians bears the name Tetrapoda.

Anderson et al. (2015) reported a number of isolated anthracosaur [PN] (embolomere or eoherpetid) bones from a mid-Tournaisian site (the Tournaisian preceded the Viséan and began at the Devonian/Carboniferous boundary 358.9 ± 0.4 Ma ago: ICSC). Whether these are tetrapods depends on the relative positions of temnospondyls, anthracosaurs and other clades in that region of the tree (Pardo et al., 2018, 2020; Marjanović and Laurin, 2019; Ruta et al., 2020; and references in all four) in addition to the position of Lissamphibia: even if the lissamphibians are temnospondyls, the anthracosaurs may still be stem-stegocephalians.

The same site has also yielded the oldest colosteid remains (Anderson et al., 2015). Colosteidae (“Colosteida” of Pardo et al., 2020) was referred to Temnospondyli throughout the 20th century and found in that position by Marjanović and Laurin (2019) to our great surprise (also in some of the trees by Daza et al., 2020: supplementary figure S15); as pointed out by Pardo et al. (2020), this means it could belong to Tetrapoda. However, ongoing work on enlarging and improving the matrix of Marjanović and Laurin (2019) and Daza et al. (2020) shows that this result was most likely an artifact of the taxon and character sample; similarly, Ruta et al. (2020) found the colosteid they included to be a temnospondyl with weak support in their Bayesian analysis, but to lie rootward of Temnospondyli in their parsimony analyses (unweighted, reweighted, or with implied weighting).

The same site has further yielded tetrapod trackways, some of which are tetradactyl (Smithson et al., 2012, and references therein). Among Paleozoic tetrapods, tetradactyly is only known among “microsaurs” (including lysorophians), scincosaurids, some urocordylids, temnospondyls, and *Colosteus* (but not its close pentadactyl relative *Greererpeton*). (Reports of tetradactyl limbs in diplocaulids have been erroneous: Marjanović and Laurin, 2019; Milner, 2019, and references therein.) *Colosteus* and probably (Clack et al., 2019) the urocordylids are stem-stegocephalians, but both were fully aquatic, thus unlikely to leave trackways; “microsaurs” and probably scincosaurids were tetrapods, and most were amphibious to terrestrial; temnospondyls spanned the full range of lifestyles, but see above for their phylogenetic position. In short, whether tetradactyl trackways are evidence of tetrapods in the mid-late Tournaisian remains unclear.

The oldest uncontroversial tetrapod is thus *Westlothiana* from close to the end of the Viséan (Marjanović and Laurin, 2019, and references therein, especially Smithson et al., 1994, 2012). Other stegocephalians from the same site and age may or may not be tetrapods: whether the temnospondyl *Balanerpeton* (Milner and Sequeira, 1994; Schoch and Milner, 2014) is one depends on the resolution of the abovementioned controversy about Lissamphibia; likewise, see above on the “anthracosaur-grade” (Marjanović and Laurin, 2019; Ruta et al., 2020) animals *Silvanerpeton* and *Eldeceeon*; *Ophiderpeton kirktonense* is an aïstopod, on which see above; *Kirktonecta* (Clack, 2011) is likely a tetrapod, but needs to be fully prepared or μCT-scanned before a confident assessment can be made.

Thus, the minimum age may be as young as roughly 335 Ma (mid-late Viséan) or as old as roughly 350 Ma (early-middle Tournaisian) depending on two phylogenetic problems.

The few Tournaisian stegocephalian sites discovered so far (Smithson et al., 2012; Anderson et al., 2015; Clack et al., 2016) have not yielded any uncontroversial tetrapods, temnospondyl bones, or temnospondyl footprints; thus, if the temnospondyls are stem-tetrapodomorphs, the ages of these sites (up to roughly 350 Ma) may be useful as a maximum age. However, as stressed by Pardo et al. (2020), they represent a very small region of the Carboniferous globe, so I continue (Marjanović and Laurin, 2019) to caution against this regardless of the phylogenetic issues. Rather, the richer and better studied Famennian (end-Devonian) record, which has not so far yielded close relatives of Tetrapoda but has yielded more rootward stegocephalians and other tetrapodomorphs (Marjanović and Laurin, 2019; Ahlberg and Clack, 2020; and references therein), should be used to place a soft maximum age around very roughly 365 Ma.

#### Node 106: Amniota [PN] (Pan-Mammalia [PN] – Sauropsida)

The cladogenesis that separated the total group of mammals (also called Synapsida [PN] or Theropsida: Goodrich, 1916) from the total group of diapsids including turtles (Sauropsida: Goodrich, 1916) was assigned a minimum age of 288 Ma (Artinskian, Early Permian) and a maximum age of 338 Ma (Viséan, Early Carboniferous).

This minimum age is rather puzzling. I am not aware of any doubts on the membership of *Hylonomus* in Sauropsida since its redescription by Carroll (1964), except the very vague ones presented by Graur and Martin (2004) and taken from even more outdated literature; none are mentioned in the review by Pardo et al. (2020) either. Because of its late Bashkirian age, this calibration has often been dated to 310 Ma (as discussed by Graur and Martin, 2004). Currently (ICSC), the Bashkirian is thought to have ended 315.2 ± 0.2 and begun 323.2 ± 0.4 Ma ago, and the site (Joggins, Nova Scotia) that has yielded *Hylonomus* has been dated to 317–319 Ma (Carpenter et al., 2015); thus, given the phylogenetic position of *Hylonomus* (Ford and Benson, 2019, and references therein), I suggest a minimum age of 318 Ma for this calibration.

There appears to be pan-mammalian material from the same site (Carroll, 1964; Mann et al., 2020), which has also yielded various “microsaurs” that Pardo et al. (2017) included in Sauropsida (see also Marjanović and Laurin, 2019, and Pardo et al., 2020). I should also emphasize that the next younger sauropsids and pan-mammals (and “microsaurs”) older than 288 Ma come from several sites in each following geological stage (Moscovian through Artinskian) and represent a considerable diversity; from the Moscovian alone, four sites of successive ages are known that present more or less complete skeletons of uncontroversial amniotes, namely, sauropsids closely related to Diapsida and *Hylonomus* (*Anthracodromeus*, *Brouffia*, *Cephalerpeton*, *Paleothyris*), the oldest “parareptile” (*Carbonodraco*) as well as what appears to be the sister-group to most other sauropsids (*Coelostegus*), and, on the pan-mammalian side, ophiacodontids (*Echinerpeton*; *Archaeothyris* from two sites). A fifth site preserves the oldest varanopid, a group of amniotes of unclear phylogenetic position (Ford and Benson, 2018, 2019). As reviewed in detail by Pardo et al. (2020), this implies ghost lineages for several other amniote clades that might not have lived in coal swamps; several of these show up in the fossil record of the next and last two stages of the Carboniferous, which ended 298.9 ± 0.15 Ma ago (ICSC). For more information on the Carboniferous amniote record, see Reisz and Modesto (1996: figure 3), Müller and Reisz (2006), Maddin et al. (2019), Mann and Paterson (2019), Mann et al. (2019), and Pardo et al. (2020), the second and the third with phylogenetic analyses, as well as references in all six. Additionally, the oldest known diadectomorphs (“diadectamorphs” of Pardo et al., 2020) date from the Kasimovian (“Missourian” in Kissel, 2010) which follows the Moscovian; they may represent the sister-group of Amniota, or they may be what should have been called non-synapsid theropsids (Klembara et al., 2019; Marjanović and Laurin, 2019; Pardo et al., 2020; and references in all three).

The absence of amniotes (and diadectomorphs) in the Serpukhovian record preceding the Bashkirian should not be given much weight for paleoecological reasons, as reviewed by Pardo et al. (2020); note that “lepospondyls” like the Viséan *Kirktonecta* and *Westlothiana*, probably closely related to but outside Amniota, are almost unknown from this age as well (candidates were described by Carroll et al., 1991; Carroll and Chorn, 1995; Lombard and Bolt, 1999). Their absence from the somewhat richer Viséan record (discussed above) suffers in part from the same problem, in part from geographic restrictions. Thus, I refrain from recommending a maximum age other than that of the preceding Node 105, even though such an early age would imply very slow rates of morphological evolution in the earliest pan-mammals and sauropsids.

#### Node 107: Reptilia [PN] (Pan-Lepidosauria [PN] – Pan-Archelosauria [PN]); Node 108: Archelosauria [PN] (Pan-Testudines [PN] – Pan-Archosauria [PN])

The origin of the sauropsid crown group by a split into Pan-Lepidosauria and Pan-Archelosauria was assigned a minimum age of 252 Ma and a maximum age of 257 Ma, both in the Late Permian. Ezcurra et al. (2014; correction: The PLOS ONE Staff, 2014) agreed that the oldest unambiguous reptile that can be clearly dated is the supposed pan-archosaur *Protorosaurus*, which is, however, 257.3 ± 1.6 Ma old as they also discussed. Therefore, they revised the minimum age to 255.7 Ma, the younger end of this confidence interval.

However, like all other recent phylogenetic analyses of molecular data, Irisarri et al. (2017) found the turtles to be closer to Archosauria [PN] than Lepidosauria [PN]. Thus, the question whether *Eunotosaurus* is a member of the turtle stem (Schoch and Sues, 2017, and references therein) becomes relevant, because the earliest occurrence of *Eunotosaurus* is roughly middle Capitanian in age (the Capitanian, the last stage of the Middle Permian, ended 259.1 ± 0.5 Ma ago and began 265.1 ± 0.4 Ma ago: ICSC), and further because *Protorosaurus* would presumably belong to Pan-Archosauria and thus calibrate Node 108, not 107.

For present purposes, I set the minimum age of Archelosauria (Node 108) as 263 Ma, the approximate midpoint of the Capitanian, and do not assign a minimum age to Reptilia (Node 107). However, in general, I have to, at our current level of understanding, recommend against using either of these nodes as a calibration. The reason are two major uncertainties about the topology of the phylogenetic tree.

First, if *Eunotosaurus* has moved from the “parareptiles” well outside Diapsida [PN]—or well inside Diapsida, though presumably still in its stem-group (Ford and Benson, 2019)—to the turtle stem within the crown group of Diapsida (i.e., Reptilia [PN]), do any other “parareptiles” follow it? The oldest known member of that assemblage, *Carbonodraco*, comes from the site of Linton in Ohio (Mann et al., 2019), which is about 307–308 Ma old (compare Reisz and Modesto, 1996; Carpenter et al., 2015), so that should be the minimum age of Archelosauria if all “parareptiles” are archelosaurs; the currently available phylogenetic analyses of “parareptiles” (Laurin and Piñeiro, 2018; MacDougall et al., 2019) have not adequately tested this question. While Schoch and Sues (2017) did test the mutual relationships of “parareptiles,” *Eunotosaurus*, and diapsids and found *Eunotosaurus* nested in the latter, several nodes away from the former, these nodes were very poorly supported. The character and taxon samples of all existing matrices for analyses of amniote phylogeny need to be substantially improved (Ford and Benson, 2018, 2019; Laurin and Piñeiro, 2018; MacDougall et al., 2019; Mann et al., 2019); Ford and Benson (2019) made a large step in that direction, but deliberately excluded *Eunotosaurus* and the turtles from their analysis so as not to have to deal with all problems at the same time.

Second, the position of *Protorosaurus* as a pan-archosaur, accepted for decades, was thrown into doubt by Simões et al. (2018), who found it as such in their Bayesian analyses of morphological or combined data (Simões et al., 2018: ext. data figures 5, 6; also, after a few changes to the dataset, Garberoglio et al., 2019: figure S2; Sobral et al., 2020: figures S9, S10), but not in their parsimony analyses of morphological data without or with implied weights (ext. data figures 3, 4; likewise Garberoglio et al., 2019: figure S3; Sobral et al., 2020: figure S7, S8), where it came out as a stem-sauropsid; the question was unresolved in their Bayesian tip-dating or tip-and-node dating analyses of combined data (ext. data figures 7, 8). After a different set of changes to the dataset, Simões et al. (2020) found *Protorosaurus* as a pan-archosaur when they used MrBayes (supplementary figures 2–5) or when they used BEAST for dating with a correction (supplementary figure 7), but not when they used BEAST for dating without a correction (supplementary figure 6). Support was moderate throughout. However, these trees are hard to compare to that of Irisarri et al. (2017) because they all find the turtles outside the diapsid crown (with limited support); no extant archosaurs or turtles, and therefore no molecular data for them, are included in these datasets. Using a smaller dataset with much denser sampling of Triassic reptiles, Pritchard et al. (2018) found *Protorosaurus* closer to Archosauria than to Lepidosauria with very strong support (parsimony bootstrap value: 100%, Bayesian posterior probability: 99.06%), but whether that is on the archosaur or the archelosaur stem could not be determined because there were no turtles in that dataset.

The maximum age of either node is likewise difficult to narrow down. Uncontroversial diapsids have a notoriously patchy Paleozoic record (Ford and Benson, 2018, and references therein); the same holds for “parareptiles,” which have only two known Carboniferous records so far (Modesto et al., 2015; Mann et al., 2019). I cannot express confidence in a maximum age other than that of Node 106, which I cannot distinguish from the maximum age of Node 105 as explained above. This leaves Node 107 without independent calibrations in the current taxon sample.

#### Node 113: Neognathae (Galloanserae [PN] – Neoaves)

The last common ancestor of *Anas*, *Gallus*, and *Meleagris* on one side and *Taeniopygia* on the other was assigned a minimum age of 66 Ma (the Cretaceous/Paleogene boundary) and a maximum age of 86.5 Ma (Coniacian/Santonian boundary, Late Cretaceous) following Benton and Donoghue (2007).

The oldest known neognath appears to be the presbyornithid stem-anserimorph (Elżanowski, 2014; Tambussi et al., 2019; within two steps of the most parsimonious trees of Field et al., 2020) *Teviornis* from somewhere low in the Late Cretaceous Nemegt Fm of Mongolia; it is known only from a carpometacarpus, two phalanges, and the distal end of a humerus that all seem to belong to the same right wing (Kurochkin et al., 2002). The most recent work on the specimen has bolstered its presbyornithid identity (De Pietri et al., 2016), even though the next younger presbyornithids are middle or late Paleocene (i.e., younger than 61.6 Ma: ICSC).

The age of the Nemegt Fm is difficult to pin down; radiometric dating of this or adjacent formations has not been possible, and the only fossils available for biostratigraphy are vertebrates that have to be compared to those of North America where marine correlations and radiometric dates are known. These comparisons favor a vaguely early Maastrichtian age, without ruling out a Campanian component. Magnetostratigraphic evidence was reported in a conference abstract by Hicks et al. (2001); I have not been able to find a follow-up publication. Hicks et al. (2001) stated that the sampled sections from the Nemegt and the conformably underlying Baruungoyot Fm “can be quite reliably correlated to the Geomagnetic Reversal Time Scale […] and clearly lie in the Campanian/Maastrichtian interval that extends from the uppermost part of subchron C33n, through chron 32 into the lower half of chron 31.” Where the Baruungoyot/Nemegt boundary lies on this scale was not mentioned. The upper boundary of the Nemegt Fm is an unconformity with a Paleocene formation.

Hicks et al. (2001) also studied the Late Cretaceous Djadokhta Fm, finding that “a distinct reversal sequence is emerging that allows us to correlate the sections in a preliminary way to the late Campanian through Maastrichtian interval that ranges from C32 to C31.” While I have not been able to find a publication by an overlapping set of authors on this finding, it agrees at least broadly with Dashzeveg et al. (2005: 18, 26, 27), whose own magnetostratigraphic work on the Djadokhta Fm indicated “that the sediments were deposited during the rapid sequence of polarity changes in the late part of the Campanian incorporating the end of Chron 33 and Chron 32 between about 75 and 71 Ma […]. However, this tentative correlation to the Geomagnetic Polarity Timescale cannot yet be certainly established.” Hasegawa et al. (2008) disagreed with the stratigraphy by Dashzeveg et al. (2005), but not with their dating.

Most often, the Djadokhta Fm has been thought to underlie the Baruungoyot Fm, but a contact between the two has not so far been identified (Dingus et al., 2008; cited without comment e.g., by Chinzorig et al., 2017); they could be partly coeval (references in Hasegawa et al., 2008). Still, it seems safe to say that most of the Nemegt Fm is younger than most of the Djadokhta Fm.

According to Milanese et al. (2018: Figure 12), the Campanian-Maastrichtian boundary (72.1 ± 0.2 Ma ago: ICSC) lies near the end of chron 32. The Djadokhta Fm thus corresponds to the end of the Campanian, the Baruungoyot Fm should have at most the same age, and the youngest magnetostratigraphic sample from the Nemegt Fm, in the earlier half of chron 31, should be about 70 Ma old. Given the stratigraphic position of *Teviornis* low within the formation and its nested phylogenetic position within Neognathae, I propose 71 Ma (within the same subchron as 70 Ma: Milanese et al., 2018: Figure 12) as the soft minimum age of the present calibration.

Field et al. (2020, p. 400) stated that the likely stem-pangallanseran “*Asteriornis* provides a firm calibration point for the minimum age of divergence of the major bird clades Galloanserae and Neoaves. We recommend that a minimum age of 66.7 million years is assigned to this pivotal neornithine node in future divergence time studies, reflecting the youngest possible age of the *Asteriornis* holotype including geochronological uncertainty.” In their supplementary information (p. 13), however, they revealed being aware of *Teviornis*, citing De Pietri et al. (2016) for its position as a presbyornithid (and thus, by their own phylogenetic analyses, an anserimorph) without discussing it any further.

Should the fragmentary *Teviornis* fall out elsewhere, the minimum age might nonetheless not have to rest on *Asteriornis*, because Vegaviidae, a clade containing the late Maastrichtian (Clarke et al., 2005; Salazar et al., 2010) *Vegavis*, *Polarornis*, and *Neogaeornis* and probably the end-Campanian (McLachlan et al., 2017) *Maaqwi*, has been found on the anserimorph stem in some of the latest analyses (Agnolín et al., 2017; Tambussi et al., 2019). However, Mayr et al. (2018) discussed reasons for skepticism, and the analyses of McLachlan et al. (2017), Bailleul et al. (2019: supplementary trees 7–11, 16, 17), Field et al. (2020), and O’Connor et al. (2020) found the vegaviids they included close to but outside Aves (or at least Galloanserae in the case of Bailleul et al., 2019; O’Connor et al., 2020, who did not sample Neoaves or Palaeognathae in the analyses in question).

As the soft maximum age, I tentatively suggest 115 Ma, an estimate of the mid-Aptian age of the terrestrial Xiagou Fm of northwestern China, which has yielded a diversity of stem-birds but no particularly close relatives of the crown (Wang et al., 2013; Bailleul et al., 2019; O’Connor et al., 2020; and references therein).

#### Node 117: Testudines [PN] (Pan-Pleurodira [PN] – Pan-Cryptodira [PN])

The origin of the turtle crown group by split into the pleurodiran [PN] and cryptodiran [PN] total groups was assigned a minimum age of 210 Ma and no maximum age; this was taken from Noonan and Chippindale (2006), who had cited a work from 1990 as their source.

The calibration dates treated above, and correspondingly in the Supplementary Material, are almost all too young (some substantially so, others by just a few million years). This one, in contrast, is far too old. It rests on the outdated interpretation of the Norian (Late Triassic) *Proterochersis* as a stem-group pan-pleurodire. With one short series of exceptions (Gaffney et al., 2006, 2007; Gaffney and Jenkins, 2010), all 21st-century treatments of Mesozoic turtle phylogeny have found *Proterochersis* and all other turtles older than those mentioned below to lie well outside the crown group (Shao et al., 2018: figures S8, S9; Sterli et al., 2019, 2020; and references therein, in Gaffney and Jenkins, 2010; Romano et al., 2014a).

The three oldest known xinjiangchelyids [PN], of which one was referred to *Protoxinjiangchelys*, seem to be between 170 and 180 Ma old (Aalenian/Bajocian boundary, Middle Jurassic, to Toarcian, late Early Jurassic; Hu et al., 2020, and reference therein). In the last 3 years, the xinjiangchelyids have been found as stem-testudinates or as stem-pan-cryptodires (Shao et al., 2018; Evers et al., 2019; González Ruiz et al., 2019: Figure 6 and supplementary figure 4; Gentry et al., 2019; Anquetin and André, 2020; Sterli et al., 2020: supplementary figure “X” = 19), even in both positions when the same matrix was analyzed with different methods (Sterli et al., 2019: Supplementary file SterlietalSupplementary_material_3.pdf).

The oldest known securely dated and securely identified crown-group turtle is thus the mid-late Oxfordian stem-pan-pleurodire *Caribemys* (de la Fuente and Iturralde-Vinent, 2001; Shao et al., 2018; mostly referred to *Notoemys* as *N. oxfordiensis* in more recent literature, e.g., Sterli et al., 2019). Given that the Oxfordian ended 157.3 ± 1.0 Ma ago (ICSC), I suggest a minimum age of 158 Ma.

The stem-pan-trionychian [PN] cryptodire *Sinaspideretes* (Tong et al., 2013), which would provide a minimum age for Cryptodira (node 118) rather than only Testudines, was long thought to have the same age or to be somewhat older. Of the three known specimens, at least one (the exact localities where the type and the other specimen were found are unknown) comes from the Upper (Shang-) Shaximiao Fm (Tong et al., 2013), which conformably overlies a sequence of two supposedly Middle Jurassic formations and is overlain by two Upper Jurassic formations (Tong et al., 2011; Xing et al., 2013), so it should be about Oxfordian to Callovian in age. The biostratigraphic evidence for the age of the Upper Shaximiao Fm is conflicting; there was no consensus on whether it is Middle or Late Jurassic (Xing et al., 2013) before Wang et al. (2018) showed that the immediately underlying Lower (Xia-) Shaximiao Fm is at most 159 ± 2 Ma old, a confidence interval that lies entirely in the Late Jurassic (which began, with the Oxfordian, 163.5 ± 1.0 Ma ago: ICSC). Most likely, then, the same holds for all *Sinaspideretes* specimens, and none of them is older than *Caribemys*.

The unambiguously Early Jurassic and Triassic record of turtles throughout Pangea lies entirely on the stem and has a rather good stratigraphic fit (see Sterli et al., 2019, 2020). I therefore suggest a soft maximum age of 185 Ma (in the Pliensbachian: ICSC) that probably postdates all of these taxa but predates the oldest possible age of the oldest known xinjiangchelyids.

#### Node 129: Toxicofera (Pan-Serpentes [PN] – Anguimorpha + Pan-Iguania [PN])

This calibration was given a minimum age of 148 Ma (Tithonian, Late Jurassic) and no maximum age. Note that the minimum age was not operational because Node 131, Iguania [PN], was given an older minimum age of 165 Ma (see Supplementary Material); in other words, Node 129 was really not calibrated at all.

Indeed, I should first mention that the pan-squamate fossil record suffers from three problems that make it difficult to calibrate this node. First, it exhibits Carroll’s Gap (Marjanović and Laurin, 2013a) very strongly. After the Middle Triassic stem-pan-squamate *Megachirella* and at least one Early Triassic pan-lepidosaur that may or may not be a pan-squamate (*Sophineta* in particular—compare the different phylogenetic analyses in Simões et al., 2018, 2020), the pan-squamate record as known today goes completely silent (see Node 131 for the one or two supposed exceptions) until the dam suddenly breaks in the Bathonian (Middle Jurassic) and representatives of the stem as well as, by current understanding, several parts of the crown appear in several sites in the northern continents and northernmost Gondwana. Second, these early representatives are all isolated and generally incomplete bones that preserve few diagnostic characters; the oldest complete skeletons come from one Tithonian (latest Jurassic) cluster of sites (Conrad, 2017), followed by a few Early Cretaceous ones as well as the oldest partially articulated material other than *Megachirella*. Third, the morphological datasets so far assembled for analysis of pan-squamate phylogeny are all so plagued by correlated characters and other problems that all of them support either Pan-Iguania as the sister-group to all other squamates, or the amphisbaenians (alone or even together with the dibamids) as the sister-group to Pan-Serpentes (e.g., Simões et al., 2020: supplementary figure 2), or both (e.g., Conrad, 2017: Figures 27, 28), while both are strongly contradicted by the molecular consensus (e.g., Irisarri et al., 2017; Garberoglio et al., 2019; Simões et al., 2020: supplementary figures 1, 3, 5, 8; Sobral et al., 2020: figure S10).

[As I try to redate the exact tree topology of Irisarri et al. (2017), it is not relevant to the present work that interesting doubts about parts of the molecular consensus have been raised from the molecular data, most recently and thoroughly by Mongiardino Koch and Gauthier (2018), who also reviewed that issue.]

The oldest known toxicoferans appear to be represented by four isolated vertebral centra from the Anoual Fm of Morocco, which is early Bathonian in age (Haddoumi et al., 2015). These bones were assigned to “cf. *Parviraptor*” by Haddoumi et al. (2015). Other material—vertebrae and jaw fragments from Europe and North America discussed in Panciroli et al. (2020)—was originally assigned to “cf.” or “aff. *Parviraptor*,” including but not limited to the late Bathonian or earliest Callovian *Eophis*, the Kimmeridgian *Diablophis* and *Portugalophis*, and *Parviraptor* itself from around the Jurassic/Cretaceous (Tithonian/Berriasian) boundary. Traditionally regarded as representing the oldest anguimorphs, these fossils would calibrate Node 130, the split between Pan-Iguania [PN] and Anguimorpha; however, phylogenetic analyses following a redescription of much of the material have found it to constitute the oldest known pan-serpents, thus calibrating Node 129 (Caldwell et al., 2015; Martill et al., 2015; by implication Conrad, 2017; accepted without analysis by Garberoglio et al., 2019; Simões et al., 2020; Schineider Fachini et al., 2020). As the Bathonian began 168.3 ± 1.3 Ma ago and ended 166.1 ± 1.2 Ma ago, i.e., with uncertainty ranges that overlap in the middle (ICSC), the suggestion of 167 Ma by Caldwell et al. (2015) would then be a reasonable minimum age for this calibration.

Alifanov’s (2019) casual referral of *Parviraptor* to an unusually large version of Mosasauria should not be construed to contradict this: the Cretaceous aquatic squamates, mosasaurs included, are probably all pan-serpents (see below), unless they lie on the common stem of Anguimorpha and Iguania (Simões et al., 2020: supplementary figure 8, with very low support).

As mentioned, all these remains are very fragmentary, and all are disarticulated; according to a reviewer, new, apparently unpublished material shows the “parviraptorids” are not snakes, and indeed Panciroli et al. (2020) were careful not to state in the text whether they agreed with the referral to the snake stem, designating “cf. *Parviraptor* sp.” as “Squamata indet.” in their faunal list (Table 1).

The next younger record of a possible toxicoferan is the just as fragmentary Callovian *Changetisaurus*, a supposed anguimorph, though Alifanov (2019) provided reasons to doubt that it is a toxicoferan. It is followed by the several species of *Dorsetisaurus*, another assemblage of skull fragments with osteoderms from the Kimmeridgian through Berriasian of Europe and North America, that was explicitly accepted as an anguimorph by Caldwell et al. (2015) and, on different grounds, Alifanov (2019), but has not, to the best of my knowledge, been included in any phylogenetic analysis. (Older and secondary literature has often claimed that the oldest *Dorsetisaurus* specimens are 148 Ma old, but the Kimmeridgian ended 152.1 ± 0.9 Ma ago: ICSC.)

Most of the rich record of Cretaceous aquatic squamates has traditionally been referred to Anguimorpha, but more likely belongs to Pan-Serpentes (e.g., Garberoglio et al., 2019; Palci et al., 2019; Sobral et al., 2020: figure S10; Simões et al., 2020: supplementary figures 3, 4, 6, 9; and references therein). It sets in in what seems to be the Hauterivian with *Kaganaias* (Evans et al., 2006; Campbell Mekarski et al., 2019); the Hauterivian ended ∼129.4 Ma ago (ICSC, uncertainty not quantified). If neither the “parviraptorids” nor *Changetisaurus* nor *Dorsetisaurus* are accepted as toxicoferans, the minimum age of Node 129 should thus be 130 Ma. To err on the side of caution, that is the age I have used here.

Due to Carroll’s Gap (Marjanović and Laurin, 2013a), I agree with Irisarri et al. (2017) in not assigning a maximum age other than that for Node 125 (Supplementary Material).

#### Node 152: Placentalia [Atlantogenata – Boreo(eu)theria)]; Node 153: Boreo(eu)theria (Laurasiatheria – Euarchontoglires/Supraprimates)

The origin of Placentalia, the crown group of Eutheria, was given a minimum age of 95.3 Ma (Cenomanian, Late Cretaceous) and a maximum age of 113 Ma (Aptian/Albian boundary, Early Cretaceous) following Benton and Donoghue (2007). Its immediate descendant nodes were not constrained.

The minimum age rests on the assumption, commonly but not universally held in 2007, that the zhelestids are “ungulates,” i.e., belong to Placentalia, or perhaps even that the zalambdalestids are related to Glires and therefore belong to Placentalia. For a long time now, as already pointed out by Parham et al. (2011), every reinvestigation of the anatomy of these Cretaceous animals, and every phylogenetic analysis that sampled Cretaceous eutherians densely (i.e., not including Zhou et al., 2019: supplementary inf. M), has found them on the eutherian stem, often not even particularly close to Placentalia (e.g., Novacek et al., 1997; Asher et al., 2005, 2019; Wible et al., 2009; Goswami et al., 2011; Halliday et al., 2015; Manz et al., 2015; Bi et al., 2018: figures 2 and SI-1; Wang et al., 2019: ext. data figure 5; and references in Parham et al., 2011; see also Fostowicz-Frelik and Kielan-Jaworowska, 2002).

A few terminal Cretaceous (late Maastrichtian) eutherians have been attributed to Placentalia in the past. This is at best dubious for all of them. *Protungulatum* (Wible et al., 2009; Halliday et al., 2015, 2019: figure 1 contrary to the text; Manz et al., 2015: figure 2a; Wang et al., 2019: ext. data figure 5; Mao et al., 2019: figure S9) and *Gypsonictops* (Halliday et al., 2015, 2019; Manz et al., 2015: figure 2; Bi et al., 2018; Wang et al., 2019: ext. data figure 5; Mao et al., 2019: figure S9) are now placed close to but consistently outside Placentalia. *Deccanolestes*—at least if the teeth and the tarsal bones belong together—is placed far away (Goswami et al., 2011 [see there also for *Sahnitherium*]; Manz et al., 2015: figures 2 and I-1; Penkrot and Zack, 2016; Halliday et al., 2019). The single worn tooth named *Kharmerungulatum*, which had been assigned to Placentalia mostly through comparison to *Protungulatum* in the first place (Prasad et al., 2007), has more recently been found outside Placentalia as well (“Although none of the strict consensus trees supported the placement of *Kharmerungulatum* within the placental crown group, the limited dental material for this taxon proved insufficient for resolving its phylogenetic relationships, and so it was removed a posteriori from the MPTs to produce the reduced strict consensus trees.”—Goswami et al., 2011, p. 16334), specifically as an adapisoriculid like *Deccanolestes* when full molecular constraints were applied by Manz et al. (2015: figure 2b). The stylinodontid taeniodont *Schowalteria* (Fox, 2016, and references therein) belongs to a clade that survived into the Eocene; the conference abstract by Funston et al. (2020) reported that a very large phylogenetic analysis has found the group outside Placentalia.

The same reasons make it difficult to decide which of the earliest Paleocene eutherians should be accepted as securely enough identified placentals, but in any case, Williamson et al. (2019, p. 220) reported that the herbivorous periptychid *Ectoconus*, estimated to have reached about 100 kg, was “present within 0.4 Ma of the K-Pg boundary”; phylogenetic analyses have found it to be not only a placental, but a laurasiatherian—Halliday et al. (2015; regardless of constraints) found it and the other periptychids on the pholidotan stem; Halliday et al. (2019), using combined data and maximum likelihood, found a comparable result with much less resolution; Püschel et al. (2019), using a somewhat smaller matrix with, however, a focus on periptychids and new data on them, recovered them as stem-artiodactylomorphs. I therefore suggest 66 Ma, the Cretaceous/Paleogene boundary (66.021 ± 0.081 Ma: Clyde et al., 2016), as the minimum age for Node 153, the basal node of Boreoeutheria (a name apparently coined by accident by Murphy et al., 2001) or simply Boreotheria (explicitly coined by Waddell et al., 2001). For Node 152, I cannot recommend a separate minimum age.

Unambiguous placentals continue to be absent worldwide in the rich Maastrichtian record (see above as well as Halliday et al., 2016; Davies et al., 2017), and even ambiguous ones except *Gypsonictops* continue to be absent in the even richer Campanian record (although there are three isolated Turonian teeth indistinguishable from both species of *Gypsonictops*: Cohen and Cifelli, 2015; Cohen, 2017), despite the presence of stem-eutherians (all northern continents, Madagascar, and India), stem-metatherians (Asia and North America), and ecologically comparable spalacotheroids (Asia and North America), meridiolestidans (South America) and gondwanatheres (South America, Madagascar, India, and some point between the late Turonian and latest Campanian of Africa—O’Connor et al., 2019). Although the Late Cretaceous fossil record of Africa is too limited to exclude the presence of placentals, and Antarctica, Australia, and New Zealand have no known Late Cretaceous mammal record at all, biogeographic parsimony does not favor the presence of Campanian or Maastrichtian placentals on these paleocontinents (e.g., Huttenlocker et al., 2018): the closest known relatives of Placentalia come from North America, followed by Asian forms, while the Indian eutherians (discussed above) are quite distant from Placentalia and the incomplete tooth from Madagascar is similarly identified as zhelestid (Averianov et al., 2003). Neither the Cenozoic fossil record nor molecular phylogenetics suggest an African origin as most parsimonious either, let alone a more southeastern one. Therefore, I suggest the Campanian/Maastrichtian boundary, rounded to 72 Ma, as the hard maximum age for Node 152. (I cannot make a separate recommendation for Node 153.) This is more generous than the result of Halliday et al. (2016), 95% of whose reconstructions of the age of Placentalia were 69.53 Ma old or younger. The discrepancy to the published molecular ages (references in Halliday et al., 2016) is most likely due to the effects of body size (Berv and Field, 2017; Phillips and Fruciano, 2018), or perhaps other factors like generation length, on rates of molecular evolution.

At this point, readers may be wondering why I have mentioned neither the extremely large phylogenetic analysis by O’Leary et al. (2013) nor the objections by Springer et al. (2019), who wrote in their abstract that “morphological cladistics has a poor track record of reconstructing higher-level relationships among the orders of placental mammals”. It would be more accurate to say that phylogenetic analysis of morphological data has *no* track record of reconstructing the phylogeny of Placentalia, good *or* bad. To avoid long-branch attraction and long-branch repulsion, any such analysis of morphological data will have to sample the enormous and poorly understood diversity of Paleo- and Eocene eutherians very densely, which will have to entail sampling enough of the characters that unite and distinguish them without falling into the trap of accumulating redundant or otherwise correlated characters that inevitably distort the tree (Marjanović and Laurin, 2019; Sookias, 2019; Celik and Phillips, 2020; and references in all three). This is so much work, and so hard to get funded, that—at the most generous count—only three attempts at such a matrix have ever been made; I should also point out that matrices of such sizes were not computationally tractable until a few years ago, at least not in less than a few months of calculation time. The first attempt is the “phenomic” matrix by O’Leary et al. (2013); as Springer et al. (2019) pointed out repeatedly, it contains no less than 4541 characters—but several hundred of these are parsimony-uninformative (O’Leary et al., 2013), and many others are redundant, which means they represent a smaller number of independent characters of which many are weighted twice or more often. At 86 terminal taxa, almost all of which are extant, the taxon sample is hopelessly inadequate for eutherian phylogeny. It is no surprise that parts of the topology are highly implausible (e.g., the undisputed stem-whale *Rodhocetus* landing on the common ungulate [PN] stem, as pointed out by Springer et al., 2019) and that even such undisputed clades as Afrosoricida, Lipotyphla, and Artiodactyla are no longer recovered when the hundreds of soft-tissue characters, which cannot be scored for the extinct terminal taxa, are removed (Springer et al., 2019), which casts doubt on the ability of that matrix to place extinct taxa accurately. The second attempt began in the doctoral thesis of Zack (2009) and was further modified and merged with other datasets in Halliday’s doctoral thesis that culminated in the publication of Halliday et al. (2015). The taxon sample contains an appreciable number of Cretaceous and Paleocene eutherians; the character sample is of course more modest and contains, as usual for mammals, a large proportion of tooth characters, some of which might be redundant (e.g., Kangas et al., 2004; Harjunmaa et al., 2014). The further improved version (Halliday et al., 2019) suffers from the drawback that all characters were reduced to two states to make the matrix tractable by maximum-likelihood software; this throws away a lot of information (probably for no gain: Sansom et al., 2018; King, 2019). The third is that of the PalM group; funded by an enormous grant, it involves a lot of people each revising a group of Paleo- or Eocene eutherians as their doctoral thesis and contributing the gained knowledge (e.g., Napoli et al., 2017) to a growing matrix (ultimately based on that of Wible et al., 2009) that will then be evaluated for character redundancy and other issues. The only phylogenetic publications that have yet resulted are conference abstracts, of which I have cited Püschel et al. (2019) and Funston et al. (2020) above.

Springer et al. (2019) went on to claim that “Sansom and Wills (2013) showed that fossils are more likely to move stemward than crownward when they are only known for biomineralized characters.” Indeed, Sansom and Wills (2013) made that claim. They had taken 78 neontological matrices of extant animals with biomineralized tissues, deleted the data for soft-tissue characters from random taxa, and found that those taxa changed their phylogenetic position significantly more often than random, and further underwent “stemward slippage” as opposed to “crownward slippage” significantly more often than random. Deleting data from hard-tissue characters instead had no such effect. Sansom and Wills (2013) concluded that some mysterious factor causes hard-tissue characters to contain a systematically misleading signal much more often than soft-tissue characters do, and that therefore the phylogenetic positions of all taxa known only from hard tissues—in other words most animal fossils—are highly suspect of falsely appearing more rootward than they really are. Therefore, fossils assigned to various stem groups could really belong to the respective crown groups, and the minimum ages of divergence-date calibrations could be systematically too young (Sansom and Wills, 2013), just as Springer et al. (2019) believed. A much simpler explanation is available: hard-tissue characters are unreliable *specifically among extant species* because the hard-tissue anatomy of extant species is usually very poorly known. For example (Marjanović and Witzmann, 2015), the vertebrae of some of western and central Europe’s most common newt species are simply unknown to science, even after 200 years or more of research, because neontologists have focused on soft-tissue anatomy, behavior, and, more recently, the genome while treating the skeleton as an afterthought. The vertebrae of salamandrids are at least known to contain a phylogenetic signal—whether the appendicular skeleton also does is anybody’s guess at this point! As our knowledge of the skeletons of extant taxa would improve, so would, I predict, the ability of hard-tissue characters to accurately resolve the phylogenetic positions of extant taxa.

#### Node 188: Crown Group of Elasmobranchii (Selachimorpha – Batomorpha)

The origin of the elasmobranch crown group by split into Selachimorpha (sharks) and Batomorpha (rays and skates) was given a minimum age of 190 Ma (Sinemurian/Pliensbachian boundary, Early Jurassic) and no maximum age. (Note that the name Neoselachii is consistently treated in the paleontological literature as if defined by one or more apomorphies, not by tree topology; it probably applies to a clade somewhat larger, and possibly much older, than its crown group.)

Any attempt to date this cladogenesis suffers from the fact that the elasmobranch fossil record consists mostly of “the tooth, the whole tooth and nothing but the tooth” (as has often been said about the Mesozoic mammalian fossil record); scales and the occasional fin spine do occur, but more substantial remains are very rare. The shape of tooth crowns is naturally prone to homoplasy, the number of phylogenetically informative characters it offers is easily overestimated due to correlations between them (e.g., Kangas et al., 2004; Harjunmaa et al., 2014; Celik and Phillips, 2020; see node 157 in the Supplementary Material), and histological studies, which are needed to determine the states of certain characters (e.g., Andreev and Cuny, 2012; Cuny et al., 2017), have not been carried out on all potentially interesting tooth taxa.

Consequently, there is not as much interest in phylogeny among specialists of early elasmobranchs than among specialists of early mammals or early dinosaurs. This goes so far as to affect the use of terminology: Andreev and Cuny (2012) mentioned “stem selachimorphs” in the title of their work, implying that they understood Selachimorpha as a clade name, but quietly revealed it to be the name of a paraphyletic assemblage on p. 263 by stating that bundled enameloid is “diagnostic for Neoselachii exclusive of batomorphs, i.e., Selachimorpha”, and their consistent referral of Synechodontiformes (see below) to “Selachimorpha” is not necessarily a referral to the crown group—even though they called bato- and selachomorphs sister-groups in the next sentence.

A safe minimum age of 201 Ma, used here, is provided by the oldest unambiguous crown-group selachimorph, the total-group galeomorph *Agaleus*, dating from the Hettangian, apparently close to its beginning (Stumpf and Kriwet, 2019, especially figure 5, and references therein), which was the beginning of the Jurassic and happened 201.3 ± 0.2 Ma ago (ICSC); I round this down (stratigraphically up) to avoid breaching the mass extinction event at the Triassic/Jurassic boundary. The oldest batoid batomorph is only sightly younger, see Node 192 (Supplementary Material).

However, this may err very far on the side of caution. Indeed, for purposes beyond the present work, I must recommend against using the minimum age of this divergence to calibrate a timetree for at least as long as the histology of Paleozoic “shark” teeth has not been studied in much more detail in a phylogenetic context. As if by typographic error, the oldest widely accepted crown-group elasmobranch is not 190 but about 290 Ma old: the oldest fossils referred to the neoselachian *Synechodus* are four teeth of Sakmarian age (referred to *S. antiquus*, whose type tooth comes from the following Artinskian age: Ivanov, 2005; Stumpf and Kriwet, 2019), and the Sakmarian ended 290.1 ± 0.26 Ma ago (ICSC). Teeth referred to other species of *Synechodus* range into the Paleocene; *S. antiquus* is the only Permian species (Andreev and Cuny, 2012). The histology of *S. antiquus* remains unknown as of Koot et al. (2014); nonetheless, Cuny et al. (2017, p. 61) regarded *S. antiquus* as “[t]he first proven selachimorph”. Rounding up, this would suggest suggest 291 Ma as the minimum age of this calibration.

(My previous suggestion—Marjanović, 2019—to use that age as a soft minimum was incoherent, as a reviewer pointed out. A soft minimum would imply that a tail of the probability distribution of the age of this node would extend to younger ages than 291 Ma, so that an age of 290 Ma would be treated as much more probable than an age of 201 Ma. The opposite is the case: both 291 and 202 are much more probable than 290, which is younger than one potential minimum age but far older than the other. If *Synechodus antiquus* is a crown-group elasmobranch, so that 291 Ma is “the correct” minimum age, 290 is impossible; if it is not a crown-group elasmobranch, so that 201 is “correct,” 290 is so much older as to be much less probable than, say, 205 or 210.)

Potential crown-group elasmobranchs older than 291 Ma are known: Andreev and Cuny (2012) and Cuny et al. (2017, p. 69) suggested that the tooth taxa *Cooleyella* and *Ginteria* could be stem-batomorphs. The oldest known *Cooleyella* specimen dates from around the end of the Tournaisian (Richards et al., 2018), which occurred 346.7 ± 0.4 Ma ago (ICSC); *Ginteria* appeared in the following Viséan stage. Cuny et al. (2017, p. 21, p. 69) further pointed out that *Mcmurdodus*, a tooth taxon that first appeared around the Early/Middle Devonian (Emsian/Eifelian) boundary (Burrow et al., 2008), has occasionally been placed within Selachimorpha, even within Hexanchiformes in the selachimorph crown-group (Burrow et al., 2008, and references therein); they very tentatively suggested a stem-selachimorph position. Boisvert et al. (2019) wondered instead if it is a stem-chondrichthyan.

The absence of any however tentative suggestions of crown-elasmobranchs before *Mcmurdodus* in the rather rich total-group chondrichthyan microfossil record despite the traditional optimism of paleodontologists may, somewhat ironically, serve as a hard maximum age for this calibration; the ICSC places the Emsian/Eifelian boundary at 393.3 ± 1.2 Ma ago, so I suggest 395 Ma.

### Analysis Methods

Johan Renaudie (Museum für Naturkunde, Berlin) kindly performed the divergence dating using the tree (topology and uncalibrated branch lengths), the model of evolution (CAT-GTR+Γ) and clock model (lognormal autocorrelated relaxed) inferred by Irisarri et al. (2017), and the data (“nuclear test data set”: the variable sites of the 14,352 most complete amino acid positions of their “NoDP” dataset), but the calibrations presented above and in the Supplementary Material (all at once, not different subsets).

The intent was to also use the software Irisarri et al. (2017) had used (PhyloBayes, though the latest version, 4.1c: Lartillot, 2015). However, PhyloBayes is unable to treat some bounds as hard and others as soft in the same analysis; it can only treat all as soft, as Irisarri et al. (2017) had done, or all as hard. Consequently, we ran our analysis with all bounds treated as hard in order to account for the hard minima (discussed above in the section “*Materials and methods*: *Hard and soft minima and maxima”*).

The launch code for our PhyloBayes analysis is: ./pb -d ali14352.phy -T final_tree.tre -cal dm4.txt -r outgroups -bd -cat -gtr -ln -dc dm4hardDC.1 &./pb -d ali14352.phy -T final_tree.tre -cal dm4.txt -r outgroups -bd -cat -gtr -ln -dc dm4hardDC.2.

Irisarri et al. (2017) ran 100 gene-jackknifed analyses for each of their two sets of calibrations. Lacking the necessary computational resources, we only ran two analyses of the full dataset, without jackknifing. The results (Table 2 and Figure 1) are therefore less reliable, given the data, than those of Irisarri et al. (2017), but they fully suffice as a proof of concept to show that improved calibrations lead to changes to many inferred node ages.

**TABLE 2:** The ages found by Irisarri et al. (2017: supplementary table 9: last three columns) when all calibrations were used (all bounds treated as soft, mean ages averaged over 100 gene-jackknifed runs, extremes absolute over all runs), and the results obtained here with the updated calibrations (all bounds treated as hard, mean ages averaged over two runs with the full dataset, extremes absolute over both runs).

	Irisarri et al. (2017)			Present results		
	
Node number	Mean age	Younger end of 95% CI	Older end of 95% CI	Mean age	Younger end of 95% CI	Older end of 95% CI
100	460	452	465	472	467	475
101	393	383	403	390	363	415
102	437	431	440	462	453	471
103	426	420	431	435	427	445
104	412	408	418	423	420	4230
105	341	331	350	363	359	365
106	289	283	296	320	318	324
107	257	256	257	301	294	307
108	254	253	256	290	282	298
109	243	242	245	250	248	252
110	120	90	162	162	129	185
111	71	66	75	160	126	182
112	137	111	173	167	141	186
113	83	70	87	105	88	115
114	63	47	73	90	70	102
115	16	8	25	66	52	80
116	92	66	130	163	136	183
117	224	211	234	181	175	185
118	206	184	221	167	148	178
119	168	133	188	142	112	157
120	155	117	176	140	109	155
121	127	90	150	135	104	151
122	95	63	124	116	86	136
123	78	45	107	120	91	139
124	192	167	211	172	160	181
125	239	233	244	254	244	268
126	199	190	208	153	141	178
127	195	185	204	150	138	175
128	187	177	196	144	133	170
129	182	173	192	141	131	167
130	181	172	190	139	128	165
131	166	159	175	131	119	157
132	137	124	151	117	101	142
133	127	111	142	115	99	141
134	130	115	145	92	67	117
135	128	104	143	113	95	138
136	94	72	119	105	91	131
137	88	66	112	102	86	128
138	64	40	91	82	69	106
139	47	26	72	72	59	93
140	11	4	25	60	46	79
141	46	25	72	77	64	100
142	27	13	49	52	37	69
143	39	21	64	74	61	97
144	22	11	42	72	58	94
145	179	167	190	106	82	134
146	156	136	172	116	103	143
147	57	34	77	69	59	93
148	44	24	65	69	58	93
149	165	146	181	138	123	164
150	165	161	172	229	217	233
151	138	136	140	142	128	157
152	94	91	96	71	69	72
153	89	85	92	68	67	70
154	61	53	65	54	50	56
155	79	71	84	66	65	67
156	91	87	94	64	56	69
157	68	62	72	66	61	68
158	50	38	60	59	51	67
159	315	300	328	328	314	339
160	307	290	323	286	275	290
161	202	173	237	170	137	188
162	192	163	226	165	131	183
163	177	146	210	143	110	160
164	168	137	199	139	106	156
165	117	86	143	104	71	119
166	92	62	117	60	36	70
167	77	49	101	59	35	69
168	53	30	74	45	26	56
169	162	134	196	143	107	163
170	201	170	232	173	156	193
171	192	161	224	170	154	192
172	186	154	218	166	148	188
173	155	123	186	147	127	172
174	105	71	140	104	85	141
175	94	62	127	74	57	109
176	70	33	110	75	60	117
177	54	22	89	71	56	111
178	156	119	189	128	104	152
179	144	106	177	123	99	147
180	160	125	194	129	105	152
181	213	162	255	181	145	247
182	155	105	195	152	116	221
183	36	12	65	71	47	116
184	223	165	279	324	289	353
185	78	48	107	164	131	190
186	6	2	15	46	25	68
187	414	402	428	390	385	404
188	293	256	332	289	253	321
189	202	140	269	154	124	187
190	156	92	223	131	101	167
191	98	50	168	74	53	114
192	207	172	262	193	184	201
193	76	42	110	73	53	115
194	380	370	390	380	352	406
195	345	338	352	276	253	298
196	330	319	340	254	222	286
197	55	18	91	118	58	175
198	277	244	297	167	119	207

*All calibration dates are shown in Table 1. All ages are rounded to whole Ma. CI, credibility interval.*

Above, I describe phylogenetic uncertainty leading to two different minimum ages for Tetrapoda (Node 105), 335 Ma and “roughly” 350 Ma. Using the younger age results in a younger bound of 359 Ma on the 95% credibility interval of this node (mean age: 363 Ma, older bound: 365 Ma, i.e., the maximum age of the calibration: Table 2); therefore, I do not consider it necessary to set the minimum age of this node to 350 Ma and run a second analysis.

Having evaluated (in the preceding section) the inherent uncertainty of each calibration before the analyses unlike Irisarri et al. (2017), I did not cross-validate the calibrations. In the words of Pardo et al. (2020), “*a priori* assessment of the quality of *a priori* node calibrations must retain logical primacy in assessing the quality of a molecular clock”. “Reductio ad absurdum” cases aside (e.g., van Tuinen and Hedges, 2004, pp. 46–47; Waggoner and Collins, 2004; Matsui et al., 2008; Phillips et al., 2009; Ruane et al., 2010), apparent inconsistencies between calibrations should be seen as indicating not that the calibrations are wrong, but that the rate of evolution varies substantially across the tree, as already expected from other considerations (e.g., Berv and Field, 2017).

## Results and Discussion

### Bibliometry

Irisarri et al. (2017: supplementary table 8) cited 15 works as sources for their calibrations, six of them compilations made by paleontologists to help molecular biologists calibrate timetrees.

Not counting Irisarri et al. (2017) and the ICSC (which has been updated at least once a year since 2008), I cite 238 references to discuss minimum ages (mostly for the age or phylogenetic position of a potentially calibrating specimen), 27 to discuss maximum ages (mostly to argue if observed absence of a clade is reliable), and 15 for both purposes. Of the total of 280, 1 each dates to 1964, 1978, 1981, 1988, and 1991; 2 each to 1994, 1995 and 1996; 1 each to 1997 and 1998; 3 to 1999; 1 to 2000; 2 to 2001; 4 to 2002; 2 to 2003; 0 to 2004; 7 to 2005; 4 to 2006; 6 each to 2007 and 2008; 5 to 2009; 5 to 2010; 8 to 2011; 9 to 2012; 15 to 2013; 12 to 2014; 23 to 2015; 24 to 2016; 23 to 2017; 28 to 2018; 50 to 2019; 28 to 2020; 1 to 2021; and 1 was published as an accepted manuscript in 2020 and is expected to come out this year in final form. (Whenever applicable, these are the years of actual publication, i.e., public availability of the layouted and proofread work, not the year of intended publication which can be a year earlier, and not the year of print which is very often one or even two years later.) Only three of these are among the 14 used by Irisarri et al. (2017), and none of them are among the six compilations they cited.

Irisarri et al. submitted their manuscript on September 16, 2016. Assuming that half of the publications cited here that were published in 2016 came out too late to be used by Irisarri et al. (2017), the total proportion of the works cited here that would have been useful to them for calibrating their timetree but were not available amounts to 142 of 280, or 50.7%. Similarly, 252 of the works cited here, or 90%, were published since mid-2005. I conclude from this extreme “pull of the recent” that knowledge in this area has an extremely short half-life; calibration dates, therefore, cannot be taken from published compilations (including the present work) or other secondary sources, but must be checked every time anew against the current primary literature. This is time-consuming even in the digital age, much more so than I expected, and requires reading more works for context than actually end up cited (for some nodes three times as many); but there is no shortcut.

### Changes in the Calibration Dates

Of the 30 minimum ages assigned by Irisarri et al. (2017), I find only one to be accurate by the current state of knowledge, that of Batrachia (Node 160: Supplementary Material) anchored by good old *Triadobatrachus* (see Ascarrunz et al., 2016, for the latest and most thorough redescription and stratigraphy, and Daza et al., 2020, for the latest and largest phylogenetic analysis).

The minimum age of Pleurodira (Node 124: Supplementary Material), which has long been known to be 100 Ma older than Irisarri et al. (2017) set it, turns out to be copied from the calibration of a much smaller clade in Noonan and Chippindale (2006), a secondary source whose minimum age for Pleurodira was actually better by a factor of four. The minimum age of Iguanidae (Node 132: Supplementary Material) turned out to be miscopied, most likely with a typographic error, from Noonan and Chippindale (2006), who had it as 25 Ma instead of the 125 Ma of Irisarri et al. (2017)—though 25 Ma is not tenable either, but too young by at least 28 Ma.

In four more cases (Osteichthyes: Node 102 [Supplementary Material]; Reptilia: Node 107; Placentalia: Node 152; Lalagobatrachia/Bombinanura: Node 170 [Supplementary Material]), I find myself unable to assign any minimum age specific to that node. In two of these cases (Reptilia and Placentalia), the specimen previously thought to constrain that node actually constrains a less inclusive clade (Archelosauria, Node 108; Boreo(eu)theria, Node 153) that was sampled but not constrained by Irisarri et al. (2017); I have used these minimum ages to constrain the latter two nodes.

As might be expected, 15 of the minimum ages are too young, by margins ranging from 1.4 to 100 Ma or, ignoring Pleurodira, 43.25 Ma (Table 1: last two columns). Unsurprisingly, this also holds for the two nodes that Irisarri et al. (2017) did not calibrate but I did: both of them were constrained by calibrated nodes whose minimum ages were too young for these two nodes. In eight cases, including Boreo(eu)theria (Node 153), the reason is the expected one, the more or less recent discovery of previously unknown fossils (mostly before 2016); the magnitude of the resulting changes ranges from 1.4 to 11 Ma. In four more cases, including the one used by Irisarri et al. (2017) to date Osteichthyes (Node 102) but by me to date the subsequent split of Dipnomorpha and Tetrapodomorpha (Node 104: Supplementary Material), the dating of the oldest known specimens has improved by 4–16.5 Ma. The specimen used to constrain Tetrapoda (Node 105) is probably not a tetrapod, but the oldest known certain tetrapods are now nonetheless dated as roughly 5 Ma older than the minimum assigned by Irisarri et al. (2017); depending on the phylogenetic hypothesis, isolated bones or (!) footprints roughly 20 Ma older that were published in 2015 could represent the oldest tetrapods instead. The remaining six cases, including Reptilia (Node 107) and Archelosauria (Node 108) by implication, are caused by phylogenetic reassignments of previously known specimens (mostly before 2016) and have effects ranging from 4 Ma to 43.25 Ma.

The minimum ages of the remaining 13 nodes (including, accidentally, Iguanidae) are too old; the margins vary from 1 to 96 Ma. This includes the case of Toxicofera (Node 129), whose minimum age of 148 Ma assigned by Irisarri et al. (2017) was not operational as that node was in fact constrained by the minimum age of its constituent clade Iguania (Node 131: Supplementary Material), 165 Ma; both of these ages are too old—I find minimum ages of 130 Ma for Toxicofera and 72 Ma for Iguania. Interestingly, none of the changes to minimum ages are due to more precise dating. There is one case of the opposite: I have changed the minimum age of Pipidae (Node 178: Supplementary Material) from 86 to 84 Ma because the oldest known safely identified pipid, *Pachycentrata*, may be somewhat older than the Coniacian/Santonian boundary (86.3 ± 0.5 Ma ago: ICSC), but also somewhat younger, so the Santonian/Campanian boundary (83.6 ± 0.2 Ma ago: ICSC) is a safer approximation. All others are due to more or less recent findings that the oldest supposed members of the clades in question cannot, or at least cannot be confidently, assigned to these clades.

I agree with the reasoning for one of the maximum ages used by Irisarri et al. (2017), that for Archosauria (Node 109: Supplementary Material), though its numeric value had to be increased by 1 Ma due to improved dating of the Permian/Triassic boundary since the source Irisarri et al. (2017) used was published in 2005.

I find myself unable to assign a separate maximum age to 7 of the 18 remaining nodes that Irisarri et al. (2017) used maximum ages for; these nodes are only constrained by the maximum ages of more inclusive clades in my reanalysis. This includes the case of Chondrichthyes (Node 187: Supplementary Material), whose maximum age of 495 Ma assigned by Irisarri et al. (2017) was not operational as that node was in fact constrained by the maximum age of the root node, 462.5 Ma; I can likewise constrain it only by the maximum age of the root, 475 Ma. In one of these cases, the new implied maximum age is younger (by 28.5 Ma) than the previously explicit maximum; in the remainder, it is older by 27–110 Ma.

Of the remaining 11 maximum ages, six were too young by 12.5–125 Ma. In one case (the root: Gnathostomata, Node 100), the old maximum is younger than the new minimum, and in two more cases (Mammalia, Node 150, and Theria, Node 151: both Supplementary Material), phylogenetic (or, in the case of Theria, possibly stratigraphic) uncertainty is the reason; the remaining three merely show greater caution on my part in interpreting absence of evidence as evidence of absence.

The remaining five I consider too old by 3.2–93 Ma; these show greater confidence on my part in interpreting absence of evidence as evidence of absence in well-sampled parts of the fossil record. The same holds, naturally, for the six nodes that lacked maximum ages in Irisarri et al. (2017) but that I propose maximum ages for; one of these new ages, however (for Lepidosauria, Node 125: Supplementary Material), is older than the previously implied maximum age provided by the next more inclusive clade, and that by 33 Ma. The other five are 60.1 Ma to no less than 261.5 Ma younger than their previously implied equivalents.

### Changes in the Divergence Dates

Reanalyzing the data of Irisarri et al. (2017) with their methods, but using the calibration ages proposed and discussed above and treating them all as hard bounds in PhyloBayes instead of treating all as soft (see section “*Materials and methods*”: “*Hard and soft minima and maxima*” and “*Analysis methods”*), generally leads to implausibly old ages and large credibility intervals for the unconstrained nodes (Figure 1 and Table 2): e.g., the last common ancestor of chickens and turkeys (Node 115) is placed around the Cretaceous/Paleogene boundary, with a 95% credibility interval that spans half of each period, and the credibility interval of the bird crown group (Aves [PN], Node 112) spans most of the Jurassic, with a younger bound less than 10 Ma younger than the age of the distant stem-avialan *Archaeopteryx* (just over 150 Ma), while the oldest known crown-birds are less than half as old, about 71 Ma (see section “Materials and Methods”: Calibrations: Node 113).

There are exceptions, however. Most notably, the squamate radiation (nodes 126–129) is constrained only between the origin of Lepidosauria (Supplementary Material: Node 125: 244–290 Ma ago) and the origin of Toxicofera (Materials and methods: Calibrations: Node 129: minimum age 130 Ma), yet it is bunched up close to the latter date, unlike in Irisarri et al. (2017) where it was more spread out and generally older even though both calibrations were younger. For example, the unconstrained origin of Squamata [PN] (Node 126) was found to have a mean age of 199 Ma by Irisarri et al. (2017), but 153 Ma here (Table 2). The crucial difference may be that Lepidosauria did not have a maximum age, but this does not explain the very short internodes from Squamata to Iguania in my results. I should point out that the oldest likely squamate remains are close to 170 Ma old (reviewed in Panciroli et al., 2020).

In part, these implausible ages may be due to effects of body size (Berv and Field, 2017) or loosely related factors like generation length: most sampled squamates are small, while the two sampled palaeognath birds (Node 116, with an evidently spurious mean age of 163 Ma) are much larger than all sampled neognaths. This may be supported by the body size increase in snakes: their oldest sampled node (Macrostomata or Afrophidia: Node 136) is placed around the Early/Late Cretaceous boundary, followed by the origin of Endoglyptodonta (Node 138) in the Late Cretaceous, while any Late Cretaceous caenophidians (a clade containing Endoglyptodonta) remain unknown, all potential Cretaceous total-group macrostomates are beset with phylogenetic uncertainty, and considerably younger dates were found by Burbrink et al. (2020) despite the use of a mid-Cretaceous potential macrostomate as a minimum-age-only calibration. Similarly, the fact that the entire credibility interval for Supraprimates/Euarchontoglires (Node 155) was younger than its calibrated minimum age when all bounds were treated as soft in Marjanović (2019) may be due to the fact that one of the two sampled supraprimates is *Homo*, the second-largest sampled mammal and the one with the second-longest generation span.

Whelan and Halanych (2016) found that the CAT-GTR model (at least as implemented in PhyloBayes) is prone to inferring inaccurate branch lengths, especially in large datasets; this may turn out to be another cause of the results described above. The omission of the constant characters from the dataset, intended to speed up calculations (Irisarri et al., 2017), may have exacerbated this problem by leading to inaccurate model parameters (Whelan and Halanych, 2016).

It is, however, noteworthy that all terminal branches inferred here are longer, in terms of time, than in Irisarri et al. (2017).

Naturally, the changes to the calibration dates have changed the inferred ages of many calibrated nodes and the sizes of their credibility intervals. For instance, Irisarri et al. (2017) inferred a mean age of 207 Ma for Batoidea, with a 90-Ma-long 95% credibility interval that stretched from 172 Ma ago to 262 Ma ago (Node 192; Table 2); that node was calibrated with a soft minimum age set to 176 Ma, but not only was no maximum age set, no other node between there and the root node (Gnathostomata, Node 100) had a maximum age either, so that effectively the maximum age for Batoidea was that of the root node, 462.5 Ma. Following the discovery of new fossils, I have increased the hard minimum age to 184 Ma; however, out of ecological considerations, I have also introduced a hard maximum age of 201 Ma, younger than the previously inferred mean age. Naturally, the new inferred mean age is also younger: 193 Ma, with a 95% credibility interval that spans the time between the calibration dates (Table 2).

Somewhat similarly, I have increased the minimum age of Mammalia (Supplementary Material: Node 150) from 162.5 to 179 Ma following improved dating of the oldest certain mammals, increased its maximum age from 191.4 to 233 Ma to account for phylogenetic uncertainty and the limits of the Norian (middle Late Triassic) fossil record, and treated both bounds as hard. While Irisarri et al. (2017) found a mean age of 165 Ma with a credibility interval from 161 to 172 Ma, straddling the minimum age but not reaching the maximum, I find an age range that reaches the new maximum but stays far away from the new minimum (mean: 229 Ma, 95% credibility interval from 217 to 233 Ma). While the next less inclusive calibrated node (151: Theria; Supplementary Material) has an increased maximum but a barely changed minimum age, both bounds of the next more inclusive calibrated node (106: Amniota) have increased by about 30 Ma, apparently pulling the inferred age of Mammalia with them.

### Pitfalls in Interpreting the Descriptive Paleontological Literature

It is widely thought that paleontologists are particularly eager to publish their specimens as the oldest known record of some taxon. Indeed, it happens that five different species of different ages are published as the oldest record of the same taxon within 10 years. In such cases, finding a specimen that can establish a minimum age for that taxon can be as simple as finding the latest publication that makes such a claim, and that can be as simple as a Google Scholar search restricted to the last few years. However, there are harder cases; I will present two.

In the Supplementary Material, I argue for using the age of *Kopidosaurus*, about 53 million years, as the minimum age of Iguanidae (Node 132). *Kopidosaurus* was named and described from a largely complete skull by Scarpetta (2020a) in a publication where the words “oldest” and “older” do not occur at all, and “first” and “ancient” only occur in other contexts—even though Scarpetta (2019) had just published on calibration dates for molecular divergence date analyses. The reason is (S. Scarpetta, personal communication 2021) that he did not think *Kopidosaurus* was the oldest iguanid; one of the two matrices he used for phylogenetic analyses contained the 56-Ma-old *Suzanniwana*, and his analyses found it as an iguanid (Scarpetta, 2020a: supplementary information; Scarpetta, 2020b). Moreover, he was aware that the publication that named and described *Suzanniwana* (Smith, 2009a) also named and described *Anolbanolis* from the same site and age and argued that both of them—known from large numbers of isolated skull bones—were iguanids. Yet, *Anolbanolis* has never, to the best of my knowledge, been included in any phylogenetic analysis; and Conrad (2015), not mentioning *Anolbanolis* and not cited by Scarpetta (2020a, b), had found the phylogenetic position of *Suzanniwana* difficult to resolve in the analysis of a dataset that included a much larger sample of early pan-iguanians.

Smith (2009a, pp. 312–313), incidentally, did not advertise *Suzanniwana* and *Anolbanolis* as the oldest iguanids either, accepting instead at least some of the even older jaw fragments that had been described as iguanid as “surely iguanid”, explicitly so for the “highly streamworn” over-62-Ma-old *Swainiguanoides* which had been described as “the oldest North American iguanid” (Sullivan, 1982). All of that and more was considered too uncertain by DeMar et al. (2017, p. 4, file S1: 26–28), who pointed out not only how fragmentary that material was (and that some of the Cretaceous specimens more likely belong to certain other squamate clades), but also that the presence of exclusive synapomorphies with Iguanidae (if confirmed) does not mean the specimens are actually inside that crown clade—they could be on its stem. As the “oldest definitive” iguanids, Dashzeveg et al. (2005: 4) accepted *Anolbanolis*, followed by the uncontroversial *Afairiguana*, which is younger than *Kopidosaurus*; curiously, they did not mention *Suzanniwana* at all.

The conclusion that the status of *Suzanniwana* and *Anolbanolis* (let alone *Swainiguanoides* and the like) is too uncertain and that *Kopidosaurus*, nowhere advertised for that purpose, should be used to set the minimum age for Node 132 was accessible to me as an outsider to the fossil record of iguanians (or indeed squamates in general), but it took me several days of searching and reading papers and their supplementary information, and I was lucky that the two papers I overlooked (pointed out by Scarpetta, personal communication 2021) do not change this conclusion.

It took me much less effort to find that, under some phylogenetic hypotheses, the oldest known tetrapod (Materials and methods: Calibrations: Node 105 – Tetrapoda) is *Casineria*, a specimen I have studied in person and published on (Marjanović and Laurin, 2019); yet, the idea had never occurred to me or apparently anyone else in the field, even though its possibility should have been evident since 2017 and even though the phylogenetic hypotheses in question are by no means outlandish—one of them is even majoritarian.

In short, the paleontological literature is not optimized for divergence dating; the questions of what is the oldest known member of a group or when exactly that group evolved often take a back seat to understanding the anatomy, biomechanics, ecology, extinction, phylogeny, or generally speaking evolution of that group in the minds of paleontologists—paleobiologists—and this is reflected in the literature. Mining it for bounds on divergence dates is still possible, as I hope to have shown, but also rather exhausting.

## Summary and Conclusion

Irisarri et al. (2017) published the largest vertebrate timetree to date, calibrated with 30 minimum and 19 maximum ages for selected nodes (although one of each was not operational because the calibrations of other nodes set tighter constraints). With just 3 years of hindsight, only one of these dates stands up to scrutiny. Of the remaining 29 minimum ages, two had to be removed altogether, two had to be moved to previously uncalibrated nodes (with modifications to their numeric values), 15 were 4–100 Ma too young, and 13 were 1–96 Ma too old. Of the 19 maximum ages, seven had to be canceled altogether, while six were too young by 13–125 Ma, and five were too old by 3–93 Ma.

One of the minimum ages was taken from the wrong node in the cited secondary source, an earlier divergence-date analysis of molecular data (Noonan and Chippindale, 2006); another from the same source had a hundred million years added without explanation, most likely by typographic error. Only six of the 30 calibrated nodes were calibrated from primary literature. The calibration dates for seven nodes were taken from the compilation by Benton and Donoghue (2007), several from other compendia, and four from Noonan and Chippindale (2006) who had not succeeded in presenting the contemporary state of knowledge either.

Using software that was only able to treat all bounds as hard or all as soft (meaning that 2.5 or 5% of the credibility interval of each inferred node age must extend beyond the bound—younger than the minimum and older than the maximum age, where present), Irisarri et al. (2017) opted to treat all bounds as soft. For all minimum ages except one, this decision is not reproducible; it is even arguable for some of the maxima. This is not a purely theoretical problem; even the inferred mean ages of some calibrated nodes were younger than their minima in Marjanović (2019).

Redating of the tree of Irisarri et al. (2017) with the presumably improved calibrations results in many changes to the mean ages of nodes and to the sizes of their credibility intervals; not all of these changes are easily predictable.

Of the 280 references I have used to improve the calibrations, 50 were published in 2019, half of the total were published after mid-2016 [when Irisarri et al. (2017) seem to have completed the work on their manuscript], and 90% were published after mid-2005. Paleontology is a fast-moving field; secondary sources cannot keep up with the half-life of knowledge. A continually updated online compendium of calibration dates would be very useful, but the only attempt to create one (Ksepka et al., 2015) is no longer funded, has not been updated since early 2018, and had limited coverage. For the time being, each new attempt to calibrate node or tip ages will have to involve finding and studying the recent paleontological and chronostratigraphic literature on the taxa, strata, and sites in question; although the Internet has made this orders of magnitude easier, it remains labor-intensive, in part because the oldest record of a clade is often not published as such, but has to be inferred from comparing several sources on phylogeny, chronostratigraphy, and sometimes taphonomy or even phylogenetics, as I illustrate here.

I urge that such work be undertaken and sufficiently funded. Accurate and precise timetrees remain an essential component of our understanding of, for example, the model organisms that are used in biomedical research: how much they can tell us about ourselves depends on how much evolution has happened along both branches since our last common ancestor, and that is in part a function of time.

## Data Availability Statement

All datasets generated for this study are included in the article/Supplementary Material.

## Author Contributions

DM designed the experiments, gathered the data, interpreted the results, prepared the figure and the tables, and wrote the manuscript.

## Conflict of Interest

The author declares that the research was conducted in the absence of any commercial or financial relationships that could be construed as a potential conflict of interest.

## Footnotes

1 https://fossilcalibrations.org

2 https://www.phyloregnum.org/
