# Supplementary material for: The Making of Calibration Sausage Exemplified by Recalibrating the Transcriptomic Timetree of Jawed Vertebrates
Source: Front Genet. 2021 May 12;12:521693. doi: 10.3389/fgene.2021.521693 (PMC8149952; doi:10.3389/fgene.2021.521693)
Supplement: Supplementary file 1 [file Data_Sheet_1.pdf]

Supplementary Material

Due to the space restrictions of Frontiers journals, most of the **Calibrations** subsection of the Methods section had to be moved here. All references cited here are presented in the References section of the main text.

1 Node 102: Osteichthyes [PN] (Pan-Actinopterygii [PN] – Sarcopterygii)

Irisarri et al. (2017) assigned a minimum age of 416 Ma and a maximum age of 439 Ma, spanning the Silurian-Devonian boundary, to the cladogenesis that created the osteichthyan crown-group by separating the sister-groups Pan-Actinopterygii and Sarcopterygii.

The minimum age of this event depends on the phylogenetic position of the "psarolepids" (Choo et al., 2017) *Guiyu* and *Sparalepis* from the Kuanti [Guandi] Fm of Yunnan, China, which represents an early part of the abovementioned Ludfordian stage (425.6 ± 0.9 to 423.0 ± 2.3 Ma ago: ICSC). The "psarolepids" lie either just outside Osteichthyes or just inside, on the sarcopterygian side of the basal dichotomy (Clement et al., 2018, and references therein). To some extent the result depends on the analysis method: Clement et al. (2018) found the "psarolepids" outside Osteichthyes by parsimony (bootstrap support throughout the tree artificially low due to missing data), but inside by Bayesian inference (94% posterior probability). Following the discussions of this issue in Choo et al. (2017), Lu et al. (2017) and Clement et al. (2018), and in particular the work of King (2019), I favor a stem-pan-osteichthyan position for this assemblage over a large number of unexpected reversals to a "placoderm" state.

The oldest known uncontroversial osteichthyan is the oldest known dipnomorph, *Youngolepis*, as discussed below; following the assignment of *Andreolepis* and *Lophosteus* to the osteichthyan stem (e.g. Botella et al., 2007; Chen et al., 2016), all certain or uncertain actinopterygians are Devonian or younger. Thus, the minimum age for this calibration is the same as that for the next, Node 104.

Likewise, for the same reasons as discussed under Node 104, I cannot assign a maximum age to this divergence other than that for the root node. I have, in other words, not calibrated this node, and recommend against using this cladogenetic event as a calibration date if Nodes 100 and 104 are available.

2 Node 104: Dipnomorpha – Tetrapodomorpha

The divergence of the sister-groups Dipnomorpha (the lungfish total group) and Tetrapodomorpha (the tetrapod total group) was assigned a minimum age of 408 and a maximum age of 419 Ma.

The minimum age may not contradict the age of the oldest known tetrapodomorph, *Tungsenia*, which is Pragian in age (Lu et al., 2012); the beginning of the Pragian is dated to 410.8 ± 2.8 Ma, its end to 407.6 ± 2.6 Ma (ICSC). However, the minimum age is clearly younger than the oldest known dipnomorphs. The oldest known specimens have been referred to *Youngolepis* and come from the lower part of the Xishancun Fm (Zhu and Fan, 1995). This formation is generally (e.g. Choo et al., 2017; Liu et al., 2017; and references therein) considered to represent the lower third or less of the Lochkovian stage, its bottom coinciding with the Silurian-Devonian boundary, which is currently dated to 419.2 ± 3.2 Ma (ICSC). However, Zhang et al. (2014) placed it in the middle of the

immediately preceding Přídolí stage, which began 423.0 ± 2.3 Ma ago (ICSC). Needing a single number to summarize this uncertainty, I suggest a minimum age of 420 Ma for Node 104, the divergence of Dipnomorpha and Tetrapodomorpha. (This is a revision stratigraphically downward from the 410 Ma recommended by Marjanović and Laurin, 2007.)

A maximum age is difficult to assign. The abovementioned Kuanti Fm, which is universally (Zhang et al., 2014) regarded as representing an early part of the Ludfordian stage which preceded the Přídolí, has yielded several gnathostomes, but the sample seems too small to tell whether the absence of dipno- and tetrapodomorphs is real. Only one even partial articulated gnathostome is known from any other Ludfordian site in the world (*Yealepis*, which lies on the chondrichthyan stem: Burrow and Young, 1999). Comparably rich sites older than the Ludfordian have not been discovered. I cannot recommend any particular maximum age for this calibration point, other than by implication the maximum age of the root node (475 Ma, see above).

3 Node 109: Archosauria [PN] (Crocodylotarsi – Pan-Aves [PN])

The origin of Archosauria by cladogenesis into the total groups of crocodiles and birds was given a minimum age of 243 Ma (Middle Triassic) and a maximum age of 251 Ma (Early Triassic).

The earliest securely dated known archosaur, belonging to the crocodile stem, is *Ctenosauriscus* from just before the end of the Olenëkian; several close relatives may be coeval or a little younger (Butler et al., 2011). The age of the Olenëkian/Anisian (Early/Middle Triassic) boundary is given in the ICSC as 247.2 Ma without a confidence interval; any such confidence interval cannot be long, however, because an Olenëkian sample has been dated to 247.32 ± 0.08 Ma, while an Anisian sample has been dated to 247.08 ± 0.11 Ma (Maron et al., 2018). Given the highly nested phylogenetic position of *Ctenosauriscus* in Archosauria (Butler et al., 2011; Ezcurra et al., 2020: ext. data fig. 4, 8), I propose 248 Ma as the minimum age of this calibration.

I accept the Permian-Triassic boundary (251.902 ± 0.024 Ma: ICSC; rounded to 252) as the soft maximum age on the grounds that a major radiation of pan-archosaurs at the beginning of the Triassic seems likely for ecological reasons: the Permian record, up to its very end, is full of pan-mammals that seem ecologically comparable to Triassic archosaurs, and given the Pangea situation of the time it seems reasonably unlikely that archosaurs existed in unsampled localities. I must caution, however, that the fossil record of pan-archosaurs and possible pan-archosaurs in the four million years of the Triassic preceding the minimum age, and in the Permian, is very patchy, with a poor fit between stratigraphy and phylogeny; indeed, the Permian record of archosauriforms [PN] is currently entirely limited to the poorly known non-archosaur *Archosaurus* and possibly the even more poorly known non-archosaur *Eorasaurus* (Ezcurra et al., 2014).

4 Node 111: Alligatoridae (Alligatorinae – Caimaninae)

The origin of Alligatoridae (the crown group of Globidonta) by split into Alligatorinae and Caimaninae was given a minimum age of 66 Ma (the Cretaceous/Paleogene boundary) and a maximum age of 75 Ma (Campanian, Late Cretaceous).

The minimum age would fit well with the finding by Cossette and Brochu (2018) that *Bottosaurus* from the very end of the Cretaceous is a caimanine. Given, however, the limited material and the stratigraphic gap between *Bottosaurus* and the next younger known caimanines, Cossette and Brochu (2018) expressed doubt about the result of their phylogenetic analysis which placed *Bottosaurus* not

only within the caimanine crown-group but next to the extant *Paleosuchus*. Cossette and Brochu (2020) did not include *Bottosaurus* in their phylogenetic analysis.

If *Bottosaurus* is not an alligatorid at all, the oldest known member is the alligatorine *Navajosuchus* from within the first million years of the Paleocene (Puercan NALMA [North American Land Mammal Age]), translating to a minimum age of 65 Ma (Wang et al., 2016, and references therein). The oldest known caimanines (*Protocaiman*, *Eocaiman paleocenicus* and *Necrosuchus*: Bona et al., 2018) follow shortly thereafter (Peligran SALMA [South American Land Mammal Age], 64–63 Ma ago: Woodburne et al., 2014).

Halliday et al. (2013), however, found the Campanian to Maastrichtian *Brachychampsa* to be an alligatorine, as did Arribas et al. (2019) in a less densely sampled analysis of Crocodyliformes; Bona et al. (2018) found it and the newly added Campanian *Albertochampsa* to be caimanines, a finding expanded by Cossette and Brochu (2020) to *Stangerochampsa*. In all these cases, the earliest record of an alligatorid is *Brachychampsa sealeyi* from early in the Campanian, which began 83.6 ± 0.2 Ma ago (ICSC). These results were not replicated by Lee and Yates (2018) or by Groh et al. (2019), who both found *Brachychampsa* on the brevirostrine stem, not as an alligatorid, and who both did not include *Albertochampsa* in their datasets. I must caution, however, that Groh et al. (2019) found Alligatorinae, and even *Alligator* itself, as a Hennigian comb in which Caimaninae was nested; this result strongly suggests that the character sample was insufficient to resolve Brevirostres.

Given this uncertainty, I have used a minimum age of 65 Ma for present purposes, but generally recommend against using this cladogenesis as a calibration for timetrees.

Up to (and including) the Campanian, the record of neosuchians is a surprisingly spotty affair (e.g. Tykoski et al., 2002; Mateus et al., 2018). Although a Late Cretaceous age of Alligatoridae (i.e. less than 100.5 Ma: ICSC) is likely, I cannot, therefore, assign a maximum age younger than the Triassic/Jurassic boundary, i.e. twice as old (201.3 ± 0.2 Ma: ICSC; rounded to 200). Only in the Triassic is the record of ecologically comparable phytosaurs dense enough to really rule out the presence of amphibious crocodylomorphs such as alligatorids. However, I have treated this maximum as hard because the likelihood that the true age approaches it is very low.

5 Node 124: Pleurodira [PN] (Pan-Chelidae – Pan-Pelomedusoides)

The origin of Pleurodira by the cladogenesis that generated Pan-Chelidae (represented by *Phrynops*) and Pan-Pelomedusoides (represented by *Pelusios*) was given a minimum age of 25 Ma (Oligocene) and no maximum age. This was miscopied from Noonan and Chippindale (2006: table 1), who assigned that age to Pelomedusidae (their calibration 18, represented here by *Pelusios* alone), not to Pleurodira; to Pleurodira they assigned (their calibration 17) a minimum age of 100 Ma (Early/Late Cretaceous boundary) and a maximum age of 150 Ma (Tithonian, Late Jurassic).

Pleurodira has long been known to extend into the Early Cretaceous (reviewed by Pérez-García, 2019); pan-podocnemidids within Pelomedusoides have a particularly rich fossil record. At present, the oldest known pleurodire is the late Barremian pan-podocnemidid *Atolchelys* (Romano et al., 2014a; Pérez-García, 2019; Hermanson et al., 2020), suggesting a minimum age of 125 Ma for this calibration (Romano et al., 2014a; ICSC).

Due to the fairly highly nested position of *Atolchelys* within Pleurodira (whether or not it is a bothremydid – Romano et al., 2014a; Cadena, 2015; Hermanson et al., 2020), and due to the somewhat sparse record of stem-pleurodires (from the Late Jurassic onwards: Romano et al., 2014a; Cadena, 2015; Pérez-García 2019), I accidentally agree with Irisarri et al. (2017) in not assigning a maximum age other than that of Node 117. The maximum age assigned by Noonan and Chippindale

(2006: table 1) "assumes the Late Jurassic *Platychelys* actually predates the origin of modern pleurodiria [sic]", which does not logically follow from the fact that it is close to but outside Pleurodira.

6 Node 125: Lepidosauria [PN] (Rhynchocephalia – Pan-Squamata [PN])

The minimum age of this calibration, given as 238 Ma, has to be slightly revised to 244 Ma (both in the Middle Triassic) based on *Megachirella*, the oldest known unambiguous stem-pan-squamate (Renesto and Bernardi, 2013; Simões et al., 2018: table S2, 2020; Garberoglio et al., 2019; Sobral et al., 2020), which is older than the oldest known rhynchocephalian (238–240 Ma: Jones et al., 2013).

The Early Triassic *Sophineta*, a large collection of isolated bones, may be a stem-pan-squamate or a stem-pan-lepidosaur (Evans and Borsuk-Białynicka, 2009a; Simões et al., 2018, 2020; Garberoglio et al., 2019; Sobral et al., 2020). The text of Sobral et al. (2020) makes clear that *Vellbergia*, another such animal, is younger than *Megachirella*, despite being shown as older in Sobral et al. (2020: fig. 4).

An Early Triassic or perhaps Late Permian maximum age seems reasonable, but, given the rarity of stem-pan-lepidosaurs and of Permian diapsids in general (Carroll's Gap – Marjanović and Laurin, 2013a), I rather propose to use the ecologically similar small amniotes (e.g. Haridy et al., 2017; MacDougall et al., 2019) of Richards Spur (up to 289.2 ± 0.68 Ma; Woodhead et al., 2010; MacDougall et al., 2017), immediately before Carroll's Gap, to support a soft maximum age of 290 Ma.

7 Node 131: Iguania [PN] (Pan-Acrodonta [PN] – Pan-Iguanidae [PN])

The origin of Iguania by cladogenesis into Pan-Acrodonta and Pan-Iguanidae was assigned a minimum age of 165 Ma (late Middle Jurassic) and a maximum age of 230 Ma (Carnian, Late Triassic) following Noonan and Chippindale (2006).

Tikiguania was described as a Late Triassic acrodontan [PN]. Not only is it an acrodontan, it is a draconine agamid (Hutchinson et al., 2012); most likely, therefore, the very well preserved isolated lower jaw is not a fossil, but belongs to one of the draconine species that live on the site, and fell into the screenwashing sample (Hutchinson et al., 2012).

Bharatagama, cited by Noonan and Chippindale (2006), is known (Evans et al., 2002) from at least 85 maxilla and dentary fragments (with supposed genuine absence of the splenial and supposed fusion of the angular to the dentary) that undoubtedly come from the Upper Member of the Kota Fm in Andhra Pradesh (India), for which, on the balance of conflicting biostratigraphic evidence (Prasad and Manhas, 2007; Prasad et al., 2014), a late Middle Jurassic age seems most likely (notwithstanding the fact that the Lower Member conformably overlies the Dharmaram Fm, which extends down into the Triassic as shown by its phytosaurs and aëtosaurs: Goswami et al., 2016). Even so, this age (i.e. 163.5 ± 1.0 Ma or older: ICSC) is old enough by comparison to the pan-iguanian fossil record and the position of Iguania in all molecular phylogenies (including Irisarri et al., 2017) that Jones et al. (2013: 15), whose molecular dating found Toxicofera as a whole to be younger than *Bharatagama*, stated: "It is possible that *Bharatagama* represents an early stem crown-group [sic] squamate with a jaw morphology convergent with modern acrodont [= acrodontan] iguanians, or that it belongs to another clade." Simões et al. (2017) cited these doubts without further comment. Evans et al. (2002: 306) listed a number of features shared by acrodontans and sphenodontians; three of these do not occur in the Cretaceous priscagamid stem-pan-acrodontans, but

all are found in *Bharatagama*. Although no known sphenodontian is a good match (Evans et al., 2002), I very tentatively suggest that *Bharatagama* could represent a morphologically innovative clade of *Diphydontosaurus*-grade sphenodontians. It would not lie outside the large (Reynoso, 2005, and references therein) sphenodontian morphospace: the shape, size, implantation and attachment of the distal teeth recalls *Clevosaurus* (depicted in Evans et al., 2002), while the shape and size of the mesial teeth is reminiscent of *Sphenovipera* (Reynoso, 2005). Indeed, the one phylogenetic analysis that has ever included *Bharatagama* found it as a rhynchocephalian rather than a squamate, although close to the pleurosaurs (despite the more *Diphydontosaurus*-like plesiomorphic gradient of tooth implantation) and, not surprisingly given the limited material, with weak support (Conrad, 2017). In sum, the optimism of Scarpetta (2019) about the usefulness of *Bharatagama* as a calibration point is unwarranted, because the status of *Bharatagama* as a pan-acrodontan is too doubtful.

Xianglong from the Yixian Fm of Liaoning (China), which dates to around the Barremian-Aptian boundary (~ 125.0 Ma: ICSC), was described as a pan-acrodontan, possibly an acrodontan (Li et al., 2007). Unfortunately, this rests on very limited evidence: the one known individual is clearly juvenile, and much of the skeleton remains unknown because is covered by exquisitely preserved soft tissue and has not been µCT-scanned (Li et al., 2007; Simões et al., 2017; Scarpetta, 2019, and reference therein).

Daza et al. (2016) briefly described three isolated hindlimbs from Burmese amber (99 Ma old: Daza et al., 2016, 2020; Wagner et al., accepted) as agamids, and a largely complete articulated individual as a chamaeleonid. The supposed chamaeleonid later turned out to be an albanerpetid amphibian with a ballistic tongue (Matsumoto and Evans, 2018: 52–53; Daza et al., 2020), and the supposed agamids are so incomplete that they probably provide more ecological than phylogenetic information; indeed, the only supposed pan-acrodontan Daza et al. (2016) included in their phylogenetic analysis was the albanerpetid. Therefore, again unlike Scarpetta (2019), I do not think any of these four specimens can be used to calibrate divergence dates.

Also from Burmese amber, Wagner et al. (accepted) described an isolated foot and lower leg that they will name *Protodraco* and offered as a calibration point for divergence dating. The scales and claws are extremely well preserved, but there is almost no evidence of bones anywhere in the specimen (Wagner et al., accepted: fig. 3). Finding only twelve informative characters in the specimen and no way to compare it to almost any other Mesozoic lepidosaur, they compared it exclusively to extant squamates in a purely phenetic principal-components analysis, which placed it closest to two agamids. Such a projection of scalation details and general proportions across a hundred million years is far too weak a basis for a calibration point. Indeed, *Protodraco* will be a nomen dubium if only because, as Wagner et al. (accepted) pointed out, the specimen cannot be compared to the priscagamids, which are known exclusively from skull material.

Priscagamidae is a Campanian clade (from the Djadokhta, Baruungoyot and more or less coeval formations; see node 113 above and Borsuk-Białynicka, 1996) of squamates that have usually been considered stem-pan-acrodontans (most recently found as such by Simões et al., 2018, and the three matrices independently derived from theirs: Garberoglio et al., 2019; Sobral et al., 2020; Simões et al., 2020; also by DeMar et al., 2017), but have also been found as stem-pan-iguanians (Conrad, 2015: fig. 6, with much denser sampling of pan-iguanians than in DeMar et al., 2017, or Simões et al., 2018, and their successors).

A consensus now appears to exist that the Campanian and Maastrichtian taxa sometimes grouped as Gobiguania (Conrad and Norell, 2007) are stem-pan-iguanians (Simões et al., 2015; Conrad, 2015), though DeMar et al. (2017: supp. inf.) could not determine if their two “gobiguanian” clades were stem-pan-iguanians or stem-pan-iguanids [PN].

"*Ctenomastax*" Gao and Norell, 2000, a junior homonym of the staphylinid beetle *Ctenomastax* Kraatz in von Heyden, 1870, is likewise known from the Djadokhta and Baruungoyot formations (see node 113); probably due to the poor preservation of the specimens (Gao and Norell, 2000), it has variously been found as the sister-group of all other pan-acrodontans (Simões et al., 2015; Reeder et al., 2015; DeMar et al., 2017) or as a gobiguanian stem-pan-iguanian (Conrad, 2015). In the latter case it cannot date the origin of Iguania.

Isodontosaurus, from the Djadokhta Fm and more or less coeval sites, is known from fairly large amounts of material representing much of the skeleton, but its phylogenetic position has been hard to determine (Gao and Norell, 2000); Conrad (2015) found it as a stem-pan-acrodontan, Reeder et al. (2015) as a gobiguanian, DeMar et al. (2017) in the "gobiguanian" grade.

DeMar et al. (2017: supp. inf.: 26–28) briefly reviewed the various Cretaceous specimens from North and South America that had been attributed to Pan-Iguanidae [PN], in some cases even Iguanidae [PN] (see node 132), and found all these attributions doubtful at best.

Alifanov (2013) described *Desertiguana* as a phrynosomatid iguanid [PN] based on an almost complete left lower jaw from the Baruungoyot Fm. Curiously, it has been summarily ignored ever since by everyone other than its author (in single-authored publications that do not provide further information and never contain phylogenetic analyses), except for a citation as a pan-iguanian without any comment by Head (2015). Given that Alifanov (2013) also classified three other Djadokhta/Baruungoyot genera otherwise considered gobiguanians as phrynosomatids, I cannot be certain that *Desertiguana* is not a gobiguanian stem-pan-iguanian as well.

Equally Campanian or older (summarized in Langer et al., 2019) is the stem-pan-acrodontan *Gueragama* (Simões et al., 2015, 2017). Known from an isolated but largely complete lower jaw, it appears to suffice for setting up a minimum age for Iguania at the Campanian/Maastrichtian boundary (72.1 ± 0.2 Ma: ICSC), which I round to 72 Ma. I should mention, however, that a reviewer doubts the phylogenetic position of *Gueragama* for unstated reasons, and that Romo de Vivar et al. (2020) found that most or all of the similarities between *Gueragama* and Acrodonta are shared with the Triassic pan-lepidosaur *Cargninia*, likely indicating that these features are evolutionarily correlated with each other and prone to convergence. Meanwhile, Alifanov (2020) called *Gueragama* an isodontosaurid (see above) without stating a reason.

Apesteguía et al. (2016) described *Jeddaherdan* from a Cenomanian jaw fragment. Using a dataset entirely restricted to iguanians, their parsimony analysis recovered it as a pan-acrodontan rather than a pan-iguanid (the only other option) and did not resolve it further until implied weighting was applied, which placed *Jeddaherdan* in a clade with *Gueragama* and the extant agamid *Uromastyx*. Bayesian inference found the same result, although with rather low support (posterior probability of 0.8). As the authors pointed out, this topology implies that the occurrence of tooth replacement in *Gueragama* is a reversal. Given the very limited material, the taxon sample which presupposes that *Jeddaherdan* is an iguanian, the constraints on the applicability of implied weighting and the poorly understood performance of Bayesian inference with missing data distributed by body part (Marjanović and Laurin, 2019, and references therein; King, 2019), as well as the implications for *Gueragama*, I prefer not to use *Jeddaherdan* to date the origin of Iguania as long as further material has not been discovered.

If none of the taxa listed above are iguanians, the fossil record of Iguania is entirely restricted to the Cenozoic, possibly beginning in the Thanetian, the last stage of the Paleocene (reviewed in Alifanov,

2020 – a work that is, however, perfectly happy to name paraphyletic taxa that are not intended as clades). I cannot assign a maximum age other than that for Node 125.

8 Node 132: Iguanidae [PN] (Iguaninae + Corytophanidae – Dactyloidae + Phrynosomatidae)

The origin of Iguanidae was given a minimum age of 125 Ma (Barremian/Aptian boundary, Early Cretaceous) and a maximum age of 180 Ma (Toarcian, Early Jurassic). This was miscopied from Noonan and Chippindale (2006), who did assign a maximum age of 180 Ma, but a minimum age of only 25 Ma (late Oligocene), citing an early Miocene specimen and its description from 1991.

Other than the Cretaceous and Paleocene questionable iguanids like *Desertiguana* (see node 131 above), it is unexpectedly hard to determine from the literature what the oldest possible iguanid could be (though even the questionable ones are all much younger than 125 Ma). Smith (2009a) described two assemblages of isolated skull bones from the Paleocene-Eocene boundary (56.0 Ma ago: ICSC) as the new taxa *Suzanniwana*, which he considered a likely stem-corytophanid, and *Anolbanolis*, which he thought close to *Polychrus* and Dactyloidae. The phylogenetic analyses by Smith (2009b: fig. 15–17) found *Suzanniwana* ("early Eocene iguanid") as a stem-corytophanid with rather low support – lower than for the polychrotid-dactyloid clade that is not supported by molecular data. Unfortunately, nobody has ever included *Anolbanolis* in a phylogenetic analysis to the best of my knowledge. DeMar et al. (2017) mentioned it in the text as one of the two oldest definitive iguanids (the other being the younger *Afairiguana*), but it does not occur in their tree figure or their entire supplementary information; *Suzanniwana* occurs nowhere in that publication at all. Conrad (2015), nowhere mentioning *Anolbanolis*, stated that *Suzanniwana* was one of the two "taxa with the most volatile positions within this analysis", but only published the Adams consensus of that analysis, which shows *Suzanniwana* as part of a polytomy that also encompasses Corytophanidae and a clade containing all other extant iguanids – whether *Suzanniwana* remains inside Iguanidae in all of the 98 most parsimonious trees or is placed as the sister-group of Iguanidae in some could only be determined by repeating the analysis. Scarpetta (2020a: supp. inf.) did include *Suzanniwana* in one of the two datasets he analyzed, and found it in the corytophanid total group or at least in a clade with Corytophanidae, *Polychrus* and Dactyloidae, but the sample of extinct species is extremely small in that matrix, and *Anolbanolis* is nowhere mentioned. Scarpetta (2020b) found *Suzanniwana* as a stem-corytophanid, a crown-corytophanid or a crown-leiosaurid (far from Corytophanidae within Iguanidae) in different model-based analyses of combined morphological and molecular data, likewise without including *Anolbanolis*, most other extinct potential iguanids or any non-iguanids other than the acrodontan outgroups, and found iguanid phylogeny difficult to resolve even with ultraconserved genomic elements due to short basal branches.

The oldest certain iguanid, then, is the oldest one known from articulated remains: the fairly highly nested *Kopidosaurus*, even though it is not clear where it is nested exactly (Scarpetta, 2020a). Being slightly older than a 52.59 ± 0.12 Ma old tuff that overlies it (Scarpetta, 2020a), and being followed by *Afairiguana* (which forms an exclusive clade with the extant *Polychrus* and Dactyloidae in the analysis of Conrad, 2015), the highly nested corytophanid *Babibasiliscus* and the less highly nested corytophanid *Geiseltaliellus* (Conrad, 2015) within the next five million years, it establishes a rather tight minimum age of 53 Ma for this calibration point, very close to the abovementioned 56 Ma.

If *Desertiguana* is not an iguanid, the absence of iguanids might suggest a late Campanian maximum age for Iguanidae. But as this possibility cannot be excluded at present, even apart from unknown geographic or ecological factors that could have kept iguanids out of the environments that deposited the Campanian and Maastrichtian formations of Asia and North America, I find myself unable to

assign a maximum age other than, again, that for Node 125. The argument by Noonan and Chippindale (2006: table 1) was "based on observations of Evans et al. (2002) and the assumption that the origin of this group does not predate the earliest known Iguaninan [sic] in the Jurassic" and is therefore doubly untenable.

Burbrink et al. (2020), Scarpetta (2020b) and references in both found extremely short internal branch lengths for the basal radiation of Iguanidae; like them, Scarpetta (2020a) found the phylogeny of Iguanidae difficult to resolve, which likewise suggests a fast radiation (but might also be a consequence of the sparse taxon sampling in both matrices). Paleoecologically, the recovery phase immediately after the Cretaceous-Paleogene boundary suggests itself as the time of such a radiation. But this remains to be tested.

9 Node 150: Mammalia [PN] (Pan-Monotremata [PN] – Theriimorpha)

The origin of the crown-group Mammalia by the divergence of Pan-Monotremata represented by *Ornithorhynchus*, on one side, and Theriimorpha, which comprises Theria (to which all extant mammals except the monotremes belong), Spalacotheroidea, Meridiolestida, Dryolestidae, Multituberculata, (Eu)triconodonta and many others, on the other side, was assigned a minimum age of 162.5 Ma (Oxfordian, Late Jurassic) and a maximum age of 191.4 Ma (Early Jurassic) following Benton and Donoghue (2007).

The phylogenetic position of Haramiyida, a clade that reaches beyond these ages into the Late Triassic, has been controversial; Celik and Phillips (2020) have presented a strong argument that it lies well outside Mammalia, which is one of the two positions found in previous analyses.

The oldest uncontroversial mammals are the pan-monotremes *Asfaltomylos* and *Henosferus* and the volaticotherian (eu)triconodont *Argentoconodon*, which all come from a level that was originally thought to be high in the Cañadón Asfalto Fm and to be Callovian or even Oxfordian (late Middle or early Late Jurassic) in age, but has more recently been correlated to its very bottom, the transition with the underlying Lonco Trapial Fm (Cúneo et al., 2013). From this bottom of the Cañadón Asfalto Fm, three successive (from lowest to highest) U-Pb zircon dates were determined by Cúneo et al. (2013): 178.766 ± 0.23 Ma, 177.37 ± 0.12 Ma and 176.15 ± 0.24 Ma. These are maximum ages in that reworked zircon crystals occur in these lacustrine tuff beds, so that the youngest crystals, from which the cited ages were derived, could still be older than the deposition of the tuff beds themselves; however, given the correlation of the recovered ages with stratigraphic height, and the rarity of older zircons in the oldest and the youngest sample (Cúneo et al., 2013), a large discrepancy is unlikely. Therefore, I recommend a minimum age of 179 Ma for this calibration.

The maximum age assigned by Irisarri et al. (2017) may be intended to represent the Sinemurian/Pliensbachian boundary (190.8 ± 1.0 Ma: ICSC). Indeed, the Sinemurian record of mammalomorphs (tritylodontids, tritheledontids, *Sinoconodon*, morganucodontans, *Hadrocodium*) from North America, southern Africa and China is fairly rich and diverse, but has not yielded mammals so far. However, ghost lineages encompassing almost the entire Early Jurassic to the middle of the Middle Jurassic occur for haramiyidans and docodonts, both of which have been found in the Rhaetian and the Bathonian, but not so far in between; and while the Rhaetian and/or possibly Norian *Thomasia* and *Haramiyavia* lie outside the smallest clade of all other haramiyidans, the Rhaetian *Tikitherium* is the sister-group of all Jurassic docodonts except the probably Middle Jurassic *Gondtherium* (Zhou et al., 2019: supp. inf. M), requiring two such ghost lineages within Docodonta. Two more such ghost lineages for Pan-Monotremata and Theriimorpha would not be very surprising. This may be especially relevant if Haramiyida, rather than the Sinemurian *Hadrocodium*, is the

sister-group of Mammalia. Currently, the former is recovered by parsimony, the latter by Bayesian analysis of the same matrix (Huttenlocker et al., 2018: extended data fig. 9; Zhou et al., 2019: supp. inf. M), neither option having strong support by its own criteria; judging from the dashes in their fig. 2 and S1, Celik and Phillips (2020) may have found the same result using an improved version of the same matrix, but they did not publish their most parsimonious trees. For comparisons between the methods as applied to paleontological datasets, see the references cited under node 102 (above). Preferring to err on the side of caution, I place the hard maximum age in the Carnian Pluvial Episode 233 Ma ago (Maron et al., 2018), which is also substantially older than all possible haramiyidans, indeed older than all currently recognized mammalomorphs (Kligman et al., 2020, and references therein).

10 Node 151: Theria (Metatheria – Eutheria)

The origin of Theria by the split into the total groups Metatheria (crown group: Marsupialia) and Eutheria (crown group: Placentalia) was given a minimum age of 124.6 Ma (Barremian/Aptian boundary, Early Cretaceous) and a maximum age of 138.4 Ma (Valanginian, Early Cretaceous) following Benton and Donoghue (2007).

The oldest securely dated therian is currently the stem-eutherian *Ambolestes* at 126 Ma (Bi et al., 2018).

Juramaia (Luo et al., 2011) has often been cited as a much older stem-eutherian. However, both its age and its phylogenetic position are in doubt; if either of these doubts is corroborated, *Juramaia* becomes irrelevant to dating this node. Originally, the only known specimen was thought to come from the Lanqi Fm, specifically a site variably called Daxigou or Daxishan (Yuan et al., 2013: supp. inf.: 4), which has meanwhile been dated to between 160.889 ± 0.069 Ma and 160.254 ± 0.045 Ma (Jia and Gao, 2019). Meng (2014: 526, 529–530), however, doubted this, called the specimen "floating", and pointed out its great similarity to *Eomaia* in particular (found as its sister-group in the very different matrices of Bi et al., 2018, and Zhou et al., 2019: supp. inf. M; Mao et al., 2019: fig. S9, did find *Juramaia* outside the clade of all other included eutherians, but did not sample *Ambolestes* despite building on the matrix of Bi et al., 2018) and to Barremian–Albian eutherians in general, as well as the long ghost lineages a mid-Oxfordian age for *Juramaia* would create within Eutheria, for Metatheria and for several of the closest relatives of Theria. Bi et al. (2018, 2019) referred to Meng (2014) for this issue but did not try to resolve it. As long as it is not resolved, I much prefer to consider the single *Juramaia* specimen to have been discovered in the Yixian Fm (like *Ambolestes*, *Eomaia* and *Acristatherium*), as suggested by Bi et al. (2019).

Celik and Phillips (2020) called *Juramaia* "purportedly Jurassic" without comment and found middling support for a sister-group relationship to Theria as a whole, noting that this agreed with earlier doubts (e.g. by Sweetman et al., 2017). However, like Mao et al. (2019), they did not sample *Ambolestes,* and the sensitivity of this result to whether parsimony or a model-based method is used was not published.

Sweetman et al. (2017) described two teeth from the very beginning of the Cretaceous (~ 145 Ma old) as two genera of Late-Cretaceous-grade eutherians, *Durlstotherium* and *Durlstodon*. In view of this limited material, I remain skeptical (see also Bi et al., 2018) and recommend 126 Ma as the minimum age for this calibration.

While the oldest uncontested metatherians are only some 110 Ma old (Bi et al., 2018), Mao et al. (2019: fig. S9) and Celik and Phillips (2020) have returned *Sinodelphys* (of the same age as *Eomaia* and *Acristatherium,* slightly younger than *Ambolestes*) to its status as the oldest known metatherian.

If this holds and if *Juramaia* has the same age instead of being Jurassic or is not a therian, and if further *Durlstotherium* and *Durlstodon* can be disregarded, virtually no ghost lineage is required at the base of Metatheria.

Accepting that *Juramaia* is not from the Lanqi Fm or not a therian, I propose 160 Ma as the soft maximum age of this calibration, on the grounds that therians or their closest relatives – other than, perhaps, *Juramaia* – are absent in the Lanqi Fm and the laterally equivalent Tiaojishan Fm, likewise absent in the Kimmeridgian and Tithonian of Portugal and the US (where the Morrison Fm, intensely sampled since the 1860s, extends across several states), and further absent in the end-Tithonian and Berriasian of England – other than, perhaps, *Durlstotherium* and *Durlstodon* – despite the diversity of ecologically comparable mammals found there. Given the strong evidence of a Laurasian origin of Theria (e.g. Huttenlocker et al., 2018; Bi et al., 2018), the earliest possible time and place for the origin of Theria that could stay out of the fossil record is therefore Asia after the deposition of the Tiaojishan and Lanqi formations ended in the Oxfordian.

11 Node 154: Carnivora [PN] (Pan-Feliformia [PN] – Pan-Caniformia [PN])

The origin of Carnivora by the divergence of the sister-groups Pan-Feliformia (represented in this matrix by *Felis*) and Pan-Caniformia (represented by *Canis*) was assigned a minimum age of 42.8 Ma (Lutetian, Eocene) and a maximum age of 63.8 Ma (Danian, Paleocene). Irisarri et al. (2017) justified this by citing the identification of the middle Eocene *Tapocyon* as a pan-caniform by Wesley and Flynn (2003); this should be regarded as rendered obsolete by Spaulding and Flynn (2012) and Solé et al. (2016), who found *Tapocyon* as a stem-carnivoriform in phylogenetic analyses of two successively larger versions of a much larger dataset. The analysis by Tomiya and Tseng (2016) found *Tapocyon* as a pan-feliform, but used a much smaller sample of stem-carnivoriforms and of characters in a misguided (e.g. Kearney and Clark, 2003; Wiens, 2003a, b, 2005a, b; Prevosti and Chemisquy, 2009; Marjanović and Laurin, 2019; King, 2019; Mongiardino Koch and Parry, 2020) attempt to avoid missing data by throwing out known data.

With “*Miacis*” *sylvestris* being recovered even more rootward on the carnivoriform stem than *Tapocyon* by Spaulding and Flynn (2012) and Solé et al. (2016), the oldest securely dated and identified carnivoran specimens belong to the amphicyonid stem-pan-caniform *Daphoenus* and the stem-canid *Hesperocyon* and are about 38 Ma old (Tomiya, 2011, and references therein). *Lycophocyon* could have the same age or be somewhat older (Tomiya, 2011), but unfortunately its phylogenetic position remains uncertain: it was published too late to be included by Spaulding and Flynn (2012), it was not added by Solé et al. (2016), and the much smaller phylogenetic analysis by Tomiya (2011) only resolved its position (as a stem-pan-caniform closer to Caniformia than *Daphoenus*) after all post-Paleogene taxa were excluded. Given the uncertainties in both age and phylogenetic position, I provisionally ignore *Lycophocyon* and suggest 38 Ma as the minimum age of this calibration.

As a hard maximum age I suggest the Paleocene/Eocene boundary 56.0 Ma ago (ICSC), around which there is a very rich record of a range of carnivorous mammals of various sizes and ecologies, including stem-carnivoriforms and many others but not including carnivorans.

12 Node 155: Euarchontoglires/Supraprimates (Gliriformes – Primatomorpha)

The last common ancestor of mice and men, the first crown-group member of a clade called Euarchontoglires (a name apparently coined by accident by Murphy et al., 2001) or, perhaps less

clunkily, Supraprimates (explicitly coined by Waddell et al., 2001), was placed between 61.5 Ma ago (Selandian, Paleocene) and 100.5 Ma ago (Early/Late Cretaceous boundary) following Benton and Donoghue (2007).

The oldest purported total-group primatomorph – not necessarily a pan-primate [PN] (Ni et al., 2016) – is *Purgatorius coracis*, found in an outcrop of the Ravenscrag Formation that is at most 0.4 Ma younger than the 66.0-Ma-old Cretaceous/Paleogene boundary (Fox and Scott, 2011; Scott et al., 2016). However, Halliday et al. (2015, 2019) found *Purgatorius* outside of Placentalia despite the presence of stem-pan-primates in their analyses. When Manz et al. (2015) applied molecular constraints (fig. 2), they did find *Purgatorius* as a pan-primate, though in a strangely nested position when the monophyly of Laurasiatheria was enforced (fig. 2b). Without constraints, the included primatomorphs formed a grade outside most other placentals (and the included laurasiatherians formed a grade outside all other placentals: fig. SI3-1). Note that Halliday et al. (2015, 2019) scored *Purgatorius* for the tarsal bones that Chester et al. (2015) referred to this taxon (somewhat younger than *P. coracis*); *Purgatorius* is otherwise known exclusively from teeth and lower jaws (Chester et al., 2015; Scott et al., 2016), and Chester et al. (2015) referred the tarsals simply because their size fits and because they show arboreal adaptations which agree with the assumed pan-primate status of *Purgatorius*. Scott et al. (2016: 343) preferred to call these bones "several isolated, possible plesiadapiform tarsals", Plesiadapiformes being a clade or grade of stem-pan-primates or stem-primatomorphs to which *Purgatorius* is generally thought to belong.

Excluding the purgatoriids, the diverse oldest known total-group primatomorphs are, in terms of North American Land Mammal Ages, slightly younger than the Puercan/Torrejonian boundary (Silcox et al., 2017), which dates to about 64.8 Ma ago (Wang et al., 2016).

On the presumably gliriform side, the oldest known members are anagalidans from the Lower Member of the Wanghudun Fm: the anagalids *Anaptogale*, *Wanogale* and *Chianshania*, the pseudictopid *Cartictops* and the astigalid *Astigale* (Missiaen et al., 2012; Wang et al., 2016; López-Torres and Fostowicz-Frelik, 2018). Their ages are poorly constrained between 66 Ma and about 62.5 Ma, though probably closer to the older end of that range (Wang et al., 2016); López-Torres and Fostowicz-Frelik (2018: fig. 4) illustrated *Anaptogale* as considerably older than *Wanogale* and *Chianshania*, but did not explain why. However, Asher et al. (2019: fig. 4, S5B, supplementary file S4-optimalTrees.nex) found Anagalida in a "primatomorph grade" when using equally weighted parsimony or implied weights with K = 24, as afrotherians with K = 2, and on the eutherian stem by Bayesian inference; at least in the latter two cases, anagalidans cannot calibrate this node.

Thus, I propose 65 Ma as the minimum age of this calibration. As the maximum age, if 66 Ma is deemed too close to the minimum (although there are presently no proposed crown- or even total-group supraprimates from the Cretaceous, despite the abundance of ecologically Glires-like and early-primatomorph-like multituberculates, gondwanatheres and the presence – in India – of ecologically pan-primate-like adapisoriculids) or to the age of the oldest *Purgatorius*, I can only offer the maximum of Node 152 (Placentalia, see above).

13 Node 157: Marsupialia (Didelphimorphia – Paucituberculata + Australidelphia)

The origin of the metatherian crown group Marsupialia was given a minimum age of 61.5 Ma (Selandian, Paleocene) and a maximum age of 71.2 Ma (Maastrichtian, Late Cretaceous) following Benton and Donoghue (2007).

Eldridge et al. (2019) reviewed this question, and found that the oldest definitive marsupials are only 54.6 Ma old as far as understood today, dating from shortly after the beginning of the Eocene (56.0

Ma ago: ICSC). Their phylogenetic and geographic position (total-group australidelphians from Australia) suggests a longer history for Marsupialia, but of the many metatherians known from the Paleocene of South America and from the Late Cretaceous through Miocene of the northern continents, none can currently be shown to belong to the crown group (Eldridge et al., 2019). I therefore propose 55 Ma as a probably overly strict minimum age for this calibration.

Carneiro (2017; not cited by Eldridge et al., 2019, whose paper was accepted for publication on 15 January 2018) found the Maastrichtian tooth taxon *Glasbius* from North America as a didelphimorphian marsupial in a phylogenetic analysis (greatly expanded from that of Carneiro and Oliveira, 2017, with the same result, likewise not cited by Eldridge et al., 2019). That analysis, however, implied an extraordinary number of transoceanic dispersals around the Paleocene and – as the Gondwanan metatherians are all Cenozoic, but most Laurasian ones are Mesozoic – a surprisingly high rate of survival of metatherians across the Cretaceous/Paleogene boundary. I must suspect that correlation, if not downright redundancy, among mammalian tooth characters has been underestimated once again (e.g. Kangas et al., 2004; Harjunmaa et al., 2014; Celik and Phillips, 2020). Indeed, Cohen et al. (2020b) found *Glasbius* on the metatherian stem; however, although they discussed this result, they did not cite Carneiro (2017) or Carneiro and Oliveira (2017). Their analysis also failed to find the two included australidelphian taxa as sister-groups despite the morphological and molecular consensus (see Eldridge et al., 2019), but the bootstrap support for this was low.

Marsupials, other metatherians and indeed other therians are wholly absent from the Late Cretaceous mammaliform record of South America, which consists instead of gondwanatherian haramiyidans, a few multituberculates and a very wide variety of meridiolestidan stem-theriiforms. The ages of the latest Cretaceous terrestrial sites of South America have been difficult to pinpoint, but there is evidence that they cover the entire Campanian and Maastrichtian (Rougier et al., 2008; Lawver et al., 2011; and references therein). The early Paleocene (Danian) sites of South America do contain stem-metatherians (and eutherians; references in Eldridge et al., 2019). If *Glasbius* is not a marsupial, it can be stated with great confidence that Marsupialia originated in South America (Eldridge et al., 2019, and references therein); if *Glasbius* is a marsupial, North America becomes the obvious candidate, and at least two clades of marsupials most likely survived the Cretaceous and immigrated into South America separately. In that case, it is noteworthy that *Glasbius* is the only possible marsupial out of the remarkable diversity of Maastrichtian, Campanian and in some cases yet earlier metatherians known from North America and to a lesser degree central Asia. Rather than the beginning of the Maastrichtian, I propose the beginning of deposition of the Lance and Hell Creek formations, where *Glasbius* has been found, as the hard maximum age for this calibration, which I estimate as 68 Ma – though the single tooth from the Williams Fork Fm that Cohen et al. (2020b) referred to *Glasbius* may be up to 2 Ma older.

14 Node 160: Batrachia (Urodela – Salientia)

The origin of Batrachia by the divergence of the sister-groups Urodela (the salamander total group now that Caudata [PN] is the crown group) and Salientia (the frog total group) was assigned a minimum age of 249 Ma and no maximum age. This was, as usual, done on the basis of *Triadobatrachus*, one of the two oldest known salientians (the other is *Czatkobatrachus*, which is probably early Olenëkian in age: Evans and Borsuk-Białynicka, 2009b); all known definitive urodeles are considerably younger (Schoch et al., 2020). Irisarri et al. (2017) only cited the classic redescription of *Triadobatrachus* from 1989 for this age; more recent stratigraphic work has been

reviewed by Ascarrunz et al. (2016: 206–207) and places *Triadobatrachus* either in the late Induan or the very beginning of the Olenëkian. Unfortunately, the precise age of the Induan-Olenëkian boundary remains unclear; the ICSC, indirectly citing a source from 2007, places it at 251.2 Ma without explicit error margins, while Maron et al. (2018) placed it at "~ 249.7 Ma" based on cyclostratigraphic counting away from the Permian-Triassic boundary, which is exceptionally precisely dated radiometrically. I conclude that 249 Ma is a perfectly adequate minimum age for this calibration point.

For a maximum age, I reiterate the suggestion of Marjanović and Laurin (2013b) to use the beginning of Carroll's Gap (see Marjanović and Laurin, 2013a), i.e. the Early Permian record, which has yielded many tetrapods ecologically comparable to batrachians, but no batrachians, gymnophionomorphs or albanerpetids so far (e.g. Schoch and Milner, 2014; Glienke, 2015). The abovementioned particularly rich site of Richards Spur, where small terrestrial and possibly amphibious temnospondyls particularly similar to batrachians are very common, has yielded three radiometric ages, of which the oldest is 289.2 ± 0.68 Ma old (Woodhead et al., 2010; MacDougall et al., 2017), so that 290 Ma may be a defensible soft maximum value. (The value of 275 Ma suggested by Marjanović and Laurin, 2007 and 2013b, is outdated.)

15 Node 169: crown group of Cryptobranchoidea (Hynobiidae – Pancryptobrancha)

The divergence between the salamander clades Pancryptobrancha (the smallest total group containing the crown group Cryptobranchidae: Vasilyan et al., 2013) and Hynobiidae was assigned a minimum age of 145.5 Ma and no maximum age.

The minimum age, intended to correspond to the Jurassic/Cretaceous boundary (~ 145.0 Ma ago: ICSC), constitutes a snapshot in the convoluted history of dating the Jurassic and Cretaceous Konservat-Lagerstätten of northeastern China. (Another such snapshot, likewise outdated, is the Valanginian age of 139.4 Ma suggested for this node by Marjanović and Laurin, 2007.) None of these sites are now considered Kimmeridgian through Valanginian in age. The oldest ones that have yielded purported caudates [PN] (references in Skutschas, 2015, beginning with Gao and Shubin, 2003, the reference cited for this calibration by Irisarri et al., 2017) belong to the Daohugou Beds, which correlate with the Haifanggou Fm and are Callovian (late Middle Jurassic) or earliest Oxfordian (Late Jurassic) in age (Gao and Shubin, 2012; Jiang et al., 2015; Liang et al., 2019; Rong et al., accepted; and references therein), not Bathonian as often claimed in older literature. These lithostratigraphic units immediately underlie the abovementioned (see node 151) Lanqi and Tiaojishan formations, which have – including in the abovementioned Daxishan or Daxigou site – likewise yielded purported caudates (Gao and Shubin, 2012; Jia and Gao, 2016, 2019).

Two Bathonian sites with supposed crown-group salamanders do exist. One (Kirtlington, UK; Forest Marble Fm) has yielded at least one undescribed vertebra called "Kirtlington salamander B". The other (Berezovsk, Russia; Itat Fm) has yielded *Kiyatriton krasnolutskii* Skutschas, 2015; while the association of the isolated bones from different body parts with each other is rather daring, the holotype of this species (like the holotype of the much younger type species, another isolated atlantal centrum) does preserve a clear synapomorphy with Caudata and three similarities to Cryptobranchoidea (Skutschas, 2014, 2015). Both sites have also yielded isolated femora that show one potential synapomorphy with Hynobiidae (Skutschas, 2014, 2015). Potentially, then, *K. krasnolutskii* could be the oldest known crown-cryptobranchoid and necessitate a minimum age of about 169 Ma (ICSC) for this node. Unfortunately, no bone referred to *Kiyatriton* has yet been included in a phylogenetic analysis, and that is not likely to happen soon: the two existing morphological datasets for analysis of salamander phylogeny (latest published versions: Wiens et al.,

2005; Rong et al., accepted) are very light on atlas characters, which moreover are mostly not accessible in the Chinese Mesozoic specimens (complete, articulated, flattened skeletons with soft-tissue outlines and melanosomes) and not well understood in extant salamanders – like the rest of the skeleton in general and the postcranial skeleton in particular, which neontologists have by and large ignored in favor of molecular, behavioral and soft-tissue characters (see Marjanović and Witzmann, 2015, for some drastic examples).

The latest published phylogenetic analysis of Mesozoic salamanders is that by Rong et al. (accepted). Like the morphological subset of Wiens et al. (2005), it produces – unless a molecular constraint is applied – a clear example of what Wiens et al. (2005: title) called "[o]ntogeny discombobulates phylogeny": a clade composed of the extant neotenic non-cryptobranchoid salamander clades, i.e. (Amphiumidae (Sirenidae, Proteidae)), as the sister-group of the metamorphic ones. Indeed, its character sample is full of characters that translate straightforwardly to presence vs. absence of a complete metamorphosis (or of a strictly aquatic lifestyle). (That is in addition to simpler, even more objective problems in the character list of the lineage of matrices from Gao and Shubin [2012] through Jia and Gao [2016, 2019] to Rong et al. [accepted]; for example, in all four of these matrices, characters 77 and 78 are duplicates of each other – the haploid number of chromosomes and the diploid number of chromosomes.) Instead, molecular data (e.g. Wiens et al., 2005; Irisarri et al., 2017; Vijayakumar et al., 2019: supplementary file Amphibia_New_India_SHL_Dryad.tre; Hime et al., 2020; and references therein) have consistently shown that Sirenidae lies outside the smallest clade formed by all other extant non-cryptobranchoid salamanders (Salamandroidea), as had long been presumed based on other considerations like the retention of external fertilization in sirenids (Reinhard et al., 2013). Likewise, Amphiumidae and Plethodontidae are consistently sister-groups in phylogenetic analyses of molecular data, rather than Amphiumidae being close to Proteidae or Sirenidae, or Plethodontidae being close to Salamandridae (e.g. Rong, 2018; Rong et al., accepted) or *Ambystoma* (e.g. Jia and Gao, 2019). This may be particularly relevant because all of the Chinese Mesozoic salamanders are either only known from larval or neotenic individuals (e.g. *Chunerpeton*: Rong et al., accepted), or are metamorphic but aquatic (*Pangerpeton*: Wang and Evans, 2006), or combine features expected of different ontogenetic stages (perhaps indicating a less condensed metamorphosis than in extant metamorphic salamanders: *Linglongtriton* [Jia and Gao, 2019]; also *Chunerpeton* [Rong et al., accepted] and, though found outside Cryptobranchoidea, *Beiyanerpeton*: Gao and Shubin, 2012), or are metamorphic and apparently terrestrial but have not been sufficiently described to be included in a phylogenetic analysis (*Laccotriton*). All known possible pancryptobranchans except the terminal Paleocene stem-pancryptobranchan *Aviturus* (Vasilyan and Böhme, 2012; Vasilyan et al., 2013) have been neotenic or undergone only partial metamorphosis (the extant *Andrias* loses the gills, the gill slits and the coronoid bone, but does not rebuild the palate or grow eyelids); this may attract stem-cryptobranchoids or even some of the more crownward stem-urodeles toward them, even if some (Rong, 2018) or most (Jia and Gao, 2019) or a variable number (Rong et al., accepted) end up in the hynobiid total group rather than in Pancryptobrancha. Unfortunately, no published phylogenetic analysis has ever included extinct Cenozoic pancryptobranchans together with any Mesozoic salamanders; the overlap between the taxon samples of Vasilyan et al. (2013) and Pearson (2016) or Rong et al. (accepted), as well as all references in all three, is restricted to extant species.

I should point out that plesiomorphies unexpected in caudates have been found in some of the Chinese Mesozoic taxa. For example, as pointed out by Marjanović and Laurin (2019: appendix S1: 76) and confirmed by Rong et al. (accepted), free palatines occur in *Chunerpeton* (Wang et al., 2015; illustrated in Gao and Shubin, 2003, though not indicated or mentioned in the text), *Beiyanerpeton*

(Gao and Shubin, 2012) and *Qinglongtriton* (Jia and Gao, 2016). This appears to be borne out by the phylogenetic analyses of Rong et al. (accepted).

It does not help that the known fossil record of possible hynobiids outside of the mentioned Lagerstätten only begins in the late Miocene and consists entirely of isolated bones (reviewed by Jia and Gao, 2016: 44–45). One possible exception is the metamorphic *Iridotriton*, known from a partial but well preserved skeleton from the early Tithonian (Galli et al., 2018; Maidment and Muxworthy, 2019) Brushy Basin Member of the Morrison Fm (Rainbow Park Microsite, Utah), originally argued to be a non-cryptobranchoid caudate (Evans et al., 2005), more recently found in an incompletely resolved position outside the cryptobranchoid crown-group (Pearson, 2016: fig. 4.11; Rong et al., accepted), and equipped with a confusing combination of characters (Evans et al., 2005).

Mesozoic pancryptobranchans seem to be represented by a large number of isolated bones from the early Cenomanian through early Campanian of Kazakhstan, Uzbekistan and Tajikistan (Skutschas, 2013) usually grouped as *Eoscapherpeton* and *Horezmia* (but considered two species of *Eoscapherpeton* by Skutschas, 2013). Unfortunately, they have never been included in a phylogenetic analysis outside of Pearson's (2016) doctoral thesis, but the arguments by Skutschas (2013) for referring at least some of the nonoverlapping material to Pancryptobrancha are not easily dismissed. In a Bayesian analysis of a matrix of morphological data containing extant lissamphibians, the Permian temnospondyls *Doleserpeton* and *Gerobatrachus*, the stem-salientian *Triadobatrachus*, *Eoscapherpeton* and a number of Cretaceous and Cenozoic scapherpetids but no other caudates, Pearson (2016: fig. 4.2) recovered *Eoscapherpeton* as a stem-pancryptobranchan, though with a posterior probability of only 52%; adding further Mesozoic salamanders led to the breakdown of this resolution (Pearson, 2016: fig. 4.12).

The oldest wholly undoubted pancryptobranchan is "*Cryptobranchus*" *saskatchewanensis*, which has been included in the phylogenetic analysis of Vasilyan et al. (2013). It comes from an exposure of the same Ravenscrag Fm that is mentioned under Node 155, but widely separated in space and age from the one mentioned there: in terms of North American Land Mammal Ages, the site with the oldest "*C.*" *saskatchewanensis* specimens – including the holotype – is Tiffanian-4 in age, thus between 59 and 60 Ma (Krause, 1978; Naylor, 1981; Wang et al., 2016: fig. 2). The material consists of isolated dentary fragments (like the holotype), maxilla fragments and an exoccipital referred by size alone; they all seem to be within the morphological range of known (Cenozoic) pancryptobranchans, but not more convincing than the similarly fragmentary *Eoscapherpeton*.

I therefore use the beginning of the Cenomanian (100.5 Ma ago, given without uncertainty in the ICSC), rounded to 101 Ma ago, as the minimum age of this calibration for present purposes. Given the great uncertainty, I generally recommend against using this divergence as a calibration.

(My previous suggestion – Marjanović, 2019 – to use this age as a soft minimum was incoherent, as a reviewer pointed out. A soft minimum would imply that a tail of the probability distribution of the age of this node would extend to younger ages than 101 Ma, so that an age of 100 Ma would be treated as much more probable than an age of, say, 61 Ma. The opposite is the case: both 101 and 60 are much more probable than 100, which is younger than one potential minimum age but far older than the other. If *Eoscapherpeton* is a crown-group cryptobranchoid, so that 101 Ma is "the correct" minimum age, 100 is impossible; if it is not a crown-group cryptobranchoid, so that 60 is "correct", 100 is so much older as to be much less probable than, say, 65.)

It is interesting in this respect that calibrating this node with an age around 139.4 Ma (Marjanović and Laurin, 2007) leads to far too high ages for cladogeneses within Hynobiidae and within

Cryptobranchidae, even within *Andrias japonicus* judging by paleogeographic criteria (Matsui et al., 2008).

Like Irisarri et al. (2017), I cannot assign a maximum age other than that of Node 160. The oldest known stem-salamanders, except for the Middle or Late Triassic *Triassurus* (Schoch et al., 2020), are Bathonian (Skutschas, 2015, and references therein); the fossil record of total-group salamanders thus exemplifies Carroll's Gap (Marjanović and Laurin, 2013a).

16 Node 170: Lalagobatrachia/Bombinanura (total group of Bombinatoroidea/Costata – total group of Pipanura); Node 171: Pipanura (total group of Pipoidea/Xenanura – total group of Acosmanura)

The last common ancestor of Bombinatoroidea or Costata, represented by *Bombina* and *Discoglossus*, and Pipanura, to which all other sampled frogs belong, was assigned a minimum age of 161.2 Ma (Oxfordian, Late Jurassic) and no maximum age. Pipanura itself was assigned a minimum age of 145.5 Ma (end-Jurassic) and no maximum age.

Following the finding that *Eodiscoglossus*, to which a Bathonian (Middle Jurassic) species has been referred that forms the basis for the original minimum age, is probably not a costatan (Báez, 2013; Báez and Gómez, 2016, 2019), the oldest purported lalagobatrachian/bombinanuran is the poorly known *Enneabatrachus* from a site dated to 152.51 ± 0.47 Ma (Trujillo et al., 2015), which has never been included in a phylogenetic analysis. Given, however, the presence of the pipanuran (rhinophrynid or stem-xenanuran: Henrici, 1998; Gómez, 2016; Aranciaga Rolando et al., 2019) *Rhadinosteus* at the same site as *Iridotriton* (the Rainbow Park Microsite, see node 169) and as further specimens of *Enneabatrachus*, a minimum age of 153 Ma for Pipanura (and Bombinanura by implication), coinciding with the maximum age of the Kimmeridgian/Tithonian boundary (152.1 ± 0.9 Ma: ICSC) and constituting a minimal revision of the age proposed by Marjanović and Laurin (2013b), appears safe.

Enneabatrachus, if not *Rhadinosteus*, is at present the oldest securely identified anuran (crown-group salientian). Remarkably, no salientians at all have so far been reported from the Yanliao Biota (Haifanggou, Lanqi, Tiaojishan and maybe other formations of Callovian to Oxfordian age in northeastern China), despite its wealth of salamanders (see node 169). The stem-salientian record is sparse (Marjanović and Laurin, 2013b; Stocker et al., 2019); the suggestion of a maximum age for Bombinanura of 170 to 185 Ma by Marjanović and Laurin (2013b) is based on the fairly good stratigraphic fit of stem-salientian phylogeny (Marjanović and Laurin, 2007, 2013a: fig. 5, 2013b; Stocker et al., 2019; and references therein), but given its poor geographic coverage, I prefer to follow Irisarri et al. (2017) in not assigning a maximum age other than that of node 160 for present purposes.

Thus, node 170 cannot currently be calibrated on its own: its minimum age is that of node 171, its maximum age is that of node 160.

17 Node 178: Pipidae (Pipinomorpha – Xenopodinomorpha)

The origin of Pipidae (the crown group of Pipimorpha) by the divergence of Pipinomorpha (crown group: Pipinae) and Xenopodinomorpha (crown group: Xenopodinae = *Xenopus* sensu lato) was given a minimum age of 86 Ma (Coniacian/Santonian boundary, Late Cretaceous) and no maximum age.

This cladogenesis is particularly difficult to date from the fossil record because molecular data support Hymenochirini as a member of Xenopodinomorpha, though less strongly than most other parts of the tree (Cannatella, 2015: fig. 1, with a bootstrap support of 71% while other branches have 74%, 93% or 100%, and with a Bayesian posterior probability of 99% while three others have 100%; Irisarri et al., 2017, with a jackknife support of 98% instead of the usual 100%; Vijayakumar et al., 2019: supplementary file Amphibia_New_India_SHL_Dryad.tre, with a Shimodaira/Hasegawa-like approximate likelihood ratio of 91% instead of the usual 100%; Hime et al., 2020: supp. fig. 4, with a bootstrap support of 100% but a local posterior measure of branch support of only 50.77% instead of the usual 80%–100%), while morphological data have so far only supported Hymenochirini as a member of Pipinomorpha (with a Bayesian posterior probability of 100% in Cannatella, 2015). The only phylogenetic analysis of combined data from pipimorphs yet undertaken (Cannatella, 2015: analysis E1) found almost equal support for both possibilities (bootstrap support of 46% vs. 44%; Bayesian posterior probabilities below 50%), and the winning-sites test could not distinguish between them (p = 1.0: Cannatella, 2015: table 3), although tip-dating with three node calibrations strongly supported the hymenochirins as pipinomorphs at the cost of losing a terminal taxon (*Pachycentrata*, see below; Cannatella, 2015: analysis E6).

Using considerably updated and expanded versions of the morphological dataset Cannatella (2015) had used, Gómez (2016), de Souza Carvalho et al. (2019) and Aranciaga Rolando et al. (2019) all found the Cenomanian *Oumtkoutia* (not included by Cannatella, 2015) to be the oldest known pipid; the Cenomanian ended 93.9 Ma ago (ICSC, no error margin given). However, while the first of these three phylogenetic analyses found it as a stem-xenopodinomorph, the other two – whose matrices are almost identical to each other, and derived from that of the first with rather few changes – found it as a stem-pipinomorph, and the third cautioned that it may well be a stem-pipimorph because, although Rage and Dutheil (2008) described the material in great detail, it consists entirely of isolated braincases, vertebrae and pelves, and there is some character conflict as *Oumtkoutia* combines a pipinomorph autapomorphy with stem-pipimorph plesiomorphies. The next younger pipid remains *Pachycentrata* of end-Coniacian or Santonian age, found as a stem-hymenochirinomorph by Gómez (2016) but as a stem-pipinomorph by de Souza Carvalho et al. (2019) and Aranciaga Rolando et al. (2019); while the Coniacian ended 86.3 ± 0.5 Ma ago, the Santonian ended only 83.6 ± 0.2 Ma ago (ICSC).

Given the presence of *Pipa* in South America but its extant sister-group Hymenochirini in Africa, and further the facts that all known pipomorphs are strictly aquatic and that lissamphibians in general tend to tolerate saltwater poorly, it is tempting to assume that this distribution is due to vicariance and the cladogenesis that separated *Pipa* and the hymenochirins should be dated to the loss of contact between Outer Gondwana (including South America) and Afro-Arabia around the Cenomanian – in other words, a geological event should be used to calibrate this divergence date. If *Pachycentrata* is a stem-hymenochirinomorph, as found by Gómez (2016), this scenario fits the phylogeny beautifully, and neither any overseas dispersal nor any long ghost lineages need to be assumed, as Gómez (2016) pointed out. Contrariwise, if *Pachycentrata* is a stem-pipinomorph, as found by de Souza Carvalho et al. (2019) and Aranciaga Rolando et al. (2019), the fossil record offers no reason to date the origin of Pipinae to the Mesozoic, and the most parsimonious hypothesis becomes that *Pipa* dispersed from Africa to South America together with the platyrrhine monkeys and the caviomorph rodents, perhaps on the same natural raft; de Souza Carvalho et al. (2019: 228) have discussed the possibility of a Paleogene island chain or even landbridge on the Walvis Ridge and the Rio Grande Rise at some length.

On the phylogenies by de Souza Carvalho et al. (2019) and Aranciaga Rolando et al. (2019), the xenopodinomorph fossil record begins only in the late Oligocene (briefly reviewed in Blackburn et al., 2019; see also Gardner and Rage, 2016: 184) rather than the Cenomanian (Gómez, 2016).

As mentioned, the only combined dataset yet brought to bear on this question (Cannatella, 2015: dataset E), which is also the only dataset containing extinct taxa that supports the hymenochirins as pipinomorphs, is based on a superseded morphological dataset that lacked *Oumtkoutia* and *Pachycentrata*, not to mention any taxa described since 2007. Given this and the discussion in the preceding paragraphs, it remains unclear whether *Oumtkoutia* is a pipid, and so I can only suggest 84 Ma as a safe minimum age for Pipidae.

Any maximum age will have to accommodate the undescribed possible pipid from the Aptian or Barremian of Cameroon (Gardner and Rage, 2016: 177, 179). However, the only maximum age I feel able to propose is much older: the end of deposition of the lake sediments of the Newark Supergroup (Tanner and Lucas, 2015) sometime around the Hettangian/Sinemurian boundary (199.3 ± 0.3 Ma ago: ICSC). All known pipimorphs, extant or extinct, have been fully aquatic (reviewed in Cannatella, 2015). The upper formations of the Newark Supergroup, which represent the rift lakes that preceded the opening of the Central Atlantic Ocean between Africa and North America, have yielded whole species flocks of semionotid actinopterygians among other parts of a lake fauna and flora (Olsen, 1988, 2010), and they cover so much space and time that if any aquatic salientians existed in northwestern Pangea during that time, we should expect to have found them – yet, salientians are consistently absent from these sediments (Olsen, 1988). The absence of salamanders (Olsen, 1988) may be explained by geography in that that group may have originated in Asia or at least northeastern Pangea (where indeed the Middle or Late Triassic *Triassurus* was found: Schoch et al., 2020). All other Barremian or earlier xenoanurans, however, have so far been found on the Iberian microcontinent or in North America, and the stratigraphic fit of their phylogeny (Gómez, 2016; Aranciaga Rolando et al., 2019) is good enough that if pipids older than *Oumtkoutia* existed, northwestern Pangea is where we should look for them. I therefore propose 199 Ma as the hard maximum age for this calibration.

It may be significant that anurans have not so far been found in the lacustrine Bathonian sediments (~ 167 Ma old) of the Anoual Fm in Morocco (Haddoumi et al., 2015).

18 Node 187: crown group of Chondrichthyes (Holocephali – Elasmobranchii)

The origin of the chondrichthyan crown group was given a minimum age of 410 Ma (Lochkovian/Pragian boundary, Devonian) and a maximum age of 495 Ma (Paibian, Furongian, Cambrian). Note that the maximum age was not operational because the root node was given a younger maximum age of 462.5 Ma.

By current understanding (Frey et al., 2019), the oldest known crown-chondrichthyan is the stem-elasmobranch *Phoebodus fastigatus* from the middle Givetian. The Givetian, part of the Middle Devonian, began 387.7 ± 0.8 Ma ago and ended 382.7 ± 1.6 Ma ago (ICSC), so I propose 385 Ma as the minimum age of the chondrichthyan crown-group.

Although I cannot assign a maximum age separate from that of the root node (node 100) to this calibration, no less than ninety million years before the minimum age, I note that this is still twenty million years after the 495 Ma assigned, futilely, by Irisarri et al. (2017).

19 Node 192: Batoidea (skates – rays)

The origin of the batomorph crown group, Batoidea, by split into skates (Rajiformes; represented by *Raja* and *Leucoraja*) and rays (taxonomically unnamed; represented by *Neotrygon*) was assigned a minimum age of 176 Ma (Toarcian, Early Jurassic) and no maximum age.

The oldest known batoid is a single rajiform tooth named *Antiquaobatis* from the late Pliensbachian, specifically the *apyrenum* subzone of the *spinatum* ammonite zone (Stumpf and Kriwet, 2019), which is close to the end of the Pliensbachian (Fraguas et al., 2018); that end occurred 182.7 ± 0.7 Ma ago (ICSC), so I propose 184 Ma as the minimum age for this calibration. (The name should of course have been "Antiquobatis", but must not be amended: ICZN, 1999: Article 32.5.1.)

As a hard maximum age, the Triassic/Jurassic boundary (201.3 ± 0.2 Ma ago: ICSC; rounded to 201 Ma) suggests itself for ecological reasons: plesiomorphically, crown-group rays are fairly large marine durophages, a guild formed by the placodont amniotes in the well sampled Middle and Late Triassic.

20 Node 195: Neopterygii [PN] (Holosteomorpha – Pan-Teleostei [PN])

The origin of Neopterygii by cladogenesis into the total groups of Holostei (bowfins – *Amia* – and gars, represented by *Lepisosteus*) and Teleostei [PN] (represented by the clupeocephalans *Takifugu* and *Danio*) was given a minimum age of 345 Ma and a maximum age of 392 Ma.

At present, there are only two candidates for Paleozoic neopterygians. One is *Acentrophorus*, "a 'semionotid'-like taxon that desperately requires restudy and formal analysis" (Friedman, 2015: 222; cited as current by Xu, 2019; also Sun et al., 2016) of Wujiapingian age (between 254.14 ± 0.07 Ma and 259.1 ± 0.5 Ma: ICSC). The "semionotids" are stem-members of Ginglymodi, i.e. closer to *Lepisosteus* than to *Amia* (Giles et al., 2017: ext. data fig. 6–8; López-Arbarello and Sferco, 2018; Xu, 2019), but a generic "'semionotid'-like taxon" could easily lie outside Neopterygii. In their in-depth study of neopterygian phylogeny, López-Arbarello and Sferco (2018) did not include *Acentrophorus* or even mention it in the text.

Sun et al. (2016) cited *Archaeolepidotus*, supposedly closely related to *Watsonulus* (see below), together with undescribed specimens as a Changxingian neopterygian (which was originally thought to be Early Triassic, but probably is not according to references in Ronchi et al., 2018). The Changxingian is the stage between the Wujiapingian and the Permian/Triassic boundary (251.902 ± 0.024 Ma ago: ICSC). *Archaeolepidotus* does not appear to be well understood; Friedman (2015), Giles et al. (2017), López-Arbarello and Sferco (2018) and Xu (2019) did not mention it, let alone include it in a phylogenetic analysis, and Google Scholar only finds 17 occurrences in the entire literature.

The oldest certain member of Neopterygii is *Watsonulus*, a stem-halecomorph or stem-holosteomorph (Friedman, 2015; Giles et al., 2017: ext. data fig. 6–8; López-Arbarello and Sferco, 2018; Xu, 2019) which comes from the Middle Sakamena Group of Madagascar (López-Arbarello and Sferco, 2018) just like *Triadobatrachus* (see node 160) and should therefore be around 249 Ma old. I therefore propose 249 Ma as the minimum age of Neopterygii.

Assuming from the almost phylogeny-free quantification of the Permo-Triassic fossil record of osteichthyans by Romano et al. (2014b) that at least the Asselian record of pan-actinopterygians [PN] is reasonably good, I suggest a soft maximum age for Neopterygii immediately before it, i.e. at the Carboniferous/Permian boundary (298.9 ± 0.15 Ma: ICSC), rounded to 299 Ma, which conveniently places it 50 Ma before the minimum age.
